# Noncovalent Interactions‐Driven Self‐Assembly of Polyanionic Additive for Long Anti‐Calendar Aging and High‐Rate Zinc Metal Batteries

**DOI:** 10.1002/advs.202404513

**Published:** 2024-06-27

**Authors:** Zimin Yang, Yilun Sun, Jianwei Li, Guanjie He, Guoliang Chai

**Affiliations:** ^1^ State Key Laboratory of Structural Chemistry Fujian Institute of Research on the Structure of Matter Chinese Academy of Sciences Fuzhou Fujian 350002 P. R. China; ^2^ College of Chemistry and Materials Science Fujian Normal University Fuzhou Fujian 350007 P. R. China; ^3^ Key Laboratory of Comprehensive and Highly Efficient Utilization of Salt Lake Resources Qinghai Province Key Laboratory of Resources and Chemistry of Salt Lakes Qinghai Institute of Salt Lakes Chinese Academy of Sciences Xining Qinghai 810008 P. R. China; ^4^ Christopher Ingold Laboratory Department of Chemistry University College London London WC1H 0AJ UK; ^5^ School of Chemical Science University of Chinese Academy of Sciences Beijing 100049 China

**Keywords:** aqueous Zn‐ion batteries, calendar aging, electrolyte additive, ion transfer, self‐assemble

## Abstract

Zinc anodes of zinc metal batteries suffer from unsatisfactory plating/striping reversibility due to interfacial parasitic reactions and poor Zn^2+^ mass transfer kinetics. Herein, methoxy polyethylene glycol‐phosphate (mPEG‐P) is introduced as an electrolyte additive to achieve long anti‐calendar aging and high‐rate capabilities. The polyanionic of mPEG‐P self‐assembles via noncovalent‐interactions on electrode surface to form polyether‐based cation channels and in situ organic–inorganic hybrid solid electrolyte interface layer, which ensure rapid Zn^2+^ mass transfer and suppresses interfacial parasitic reactions, realizing outstanding cycling/calendar aging stability. As a result, the Zn//Zn symmetric cells with mPEG‐P present long lifespans over 9000 and 2500 cycles at ultrahigh current densities of 120 and 200 mA cm^−2^, respectively. Besides, the coulombic efficiency (CE) of the Zn//Cu cell with mPEG‐P additive (88.21%) is much higher than that of the cell (36.4%) at the initial cycle after the 15‐day calendar aging treatment, presenting excellent anti‐static corrosion performance. Furthermore, after 20‐day aging, the Zn//MnO_2_ cell exhibits a superior capacity retention of 89% compared with that of the cell without mPEG‐P (28%) after 150 cycles. This study provides a promising avenue for boosting the development of high efficiency and durable metallic zinc based stationary energy storage system.

## Graphical abstract

Methoxy polyethylene glycol‐phosphate (mPEG‐P), an additive containing chain‐end phosphate and polyether polar groups, self‐assembles on the metal interface, which forms solid electrolyte interphase (SEI) along with polyether‐based channels to facilitate rapid transfer of Zn^2+^ and suppress interfacial parasitic reactions, realizing outstanding cycling/calendar aging stability and shedding light on achieving high‐efficiency energy storage system.

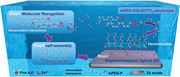

## 1 Introduction

Rechargeable non‐aqueous lithium‐ion batteries have emerged as a dominant electrochemical energy storage device for powering portable electronics and electric vehicles.^[^ ^1^ ^]^ However, their flammable and toxic electrolytes limit their application in grid‐scale energy storage.^[^ ^2^ ^]^ In contrast, aqueous zinc‐ion batteries (AZIBs) are considered one of the most promising energy storage devices for future large‐scale energy storage systems due to their low cost, high safety, and environmentally friendly characteristics, as well as their low zinc‐metal potential (−0.76 V vs SHE) and high specific capacity (820 mAh g^−1^).^[^ ^3^ ^]^ However, their commercial applications still face many challenges, mainly including corrosion of zinc anodes in resting process of batteries and rapid performance degradation under high current density during (dis)charge process. More specifically, metallic zinc electrodes will spontaneously and continuously undergo side reactions such as hydrogen evolution and corrosion in the electrolyte, especially during the resting process of batteries. This irreversibly consumes the active material and electrolyte, resulting in poor cycling/calendar aging stability of the batteries for practical applications.^[^ ^4^ ^]^ The calendar aging characteristics of batteries are directly related to their lifetime and reliability, which significantly impact the performance and cost‐efficiency of energy storage systems. Hence, eliminating the corrosion of the Zn anode is crucial to realize the commercial applications of AZIBs.

Moreover, (dis)charge process under high current densities could induce an uneven electric field and incomplete zinc stripping. Concurrently, the mass transfer of Zn^2+^ could not keep pace with the charge transfer, thereby causing concentration polarization effects and triggering short circuits within the cells.^[^ ^4c^ ^]^ To realize the safe operation of AZIBs at high current density along with high plating capacity, numerous studies have been carried out on electrolyte and electrode modification.^[^ ^5^ ^]^ The interfacial engineering of the Zn/electrolyte interphase including artificial coated and in situ formed solid electrolyte interphase (SEI) layers, which provide promising and attractive routes to improve the diffusion rate of Zn^2+^ and inhibit the side reactions. However, the artificial coated electrodes being covered with continuously deposited zinc will lead to a large volumetric change and gradually lose the protective effect from the artificial interphase.^[^ ^6^ ^]^ The in situ formed SEI layer is an effective approach to overcome the issues because of their simplicity, low cost, and controllability of dynamic interfacial regulation. Specifically, the in situ formed organic SEI layer can protect the layer from the cracking caused by the huge volume changes and facilitate the mass transfer of Zn^2+^ while suffering from poor mechanical strength.^[^ ^7^ ^]^ Furthermore, the in situ formed inorganic SEI layer can suppress water decomposition and Zn dendrite growth by preventing direct contact between Zn and water, but it generally lacks mechanical flexibility and is easily destroyed under high reversible capacity.^[^ ^8^ ^]^ Accordingly, in situ building a protective layer with hybrid ingredients will be promising for a strong SEI layer, which can more effectively improve the mass transfer of Zn^2+^ and simultaneously inhibit the corrosion reactions than those containing only inorganic or organic components. Therefore, it is imperative to delve into the two‐phase interfacial engineering design between the electrolyte and zinc anodes, improving the calendar life and high‐rate cycling performance of AZIBs.

**Scheme 1:**
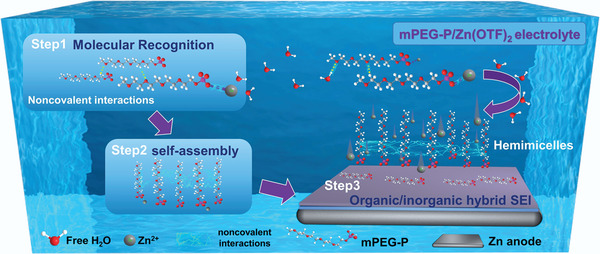
The schematic regulation diagrams of Zn deposition behaviors in mPEG‐P/Zn(OTf)_2_ electrolyte.

In this work, we introduced methoxy polyethylene glycol‐phosphate (mPEG‐P) as an electrolyte additive featuring molecular recognition and self‐assembly to form a flexible SEI with hybrid ingredients to optimize the Zn^2+^ mass transfer, which enhances the cycling performance of AZIBs under high current density and anti‐calendar aging (**Scheme** **1**). The formation of a phospholipid bilayer, the association of a protein with a ligand, and the interaction of a transcription factor with DNA are all controlled by molecular recognition, a process which is characterized as a process between molecules controlled by noncovalent interactions like electrostatic interactions, van der Waals forces, and hydrophobic effects, which serves as the foundation for self‐assembly.^[^ ^9^ ^]^ Herein, the dissociation of hydrogen from the phosphate group of mPEG‐P leads to the formation of mPEG‐P‐derived polyanions which facilitates noncovalent interactions among mPEG‐P chains, enhancing the chemisorption activity of mPEG‐P with Zn metal and regulating the deposition of Zn_x_OTf_y_(OH)_2x‐y·_ *n*H_2_O. Subsequently, mPEG‐P chains self‐assemble on the metal interface and form vertically aligned hemimicelles,^[^ ^10^ ^]^ constructing polyether‐based cation channels to endow the electrode with fast reaction kinetics. Meanwhile, in situ generation of the gradient SEI layer promotes the uniform plating of Zn^2+^ and reduces interfacial parasitic reactions, realizing excellent anti‐calendar aging performance. Similarly, dissolution of Mn^2+^ from the manganese dioxide cathode material is also significantly suppressed through the interfacial regulation for the full cell test. Consequently, to the best of our knowledge, the Zn//Zn symmetric batteries exhibit a record high performance over 9000 and 2500 cycles at ultrahigh current densities of 120 mA and 200 mA cm^−2^, respectively. Meanwhile, the symmetric cell can withstand up to an unprecedented high cumulative plating capacity of 30 mAh cm^−2^ and a current density of 30 mA cm^−2^ during rate performance measurements. With respect to the anti‐aging characterizations, the coulombic efficiency (CE) of the Zn//Cu cell with mPEG‐P additive (88.21%) is much higher than that of the cell (36.4%) in control groups at the initial cycle after 15‐day calendar aging treatment. Moreover, after 20‐day aging, the Zn//MnO_2_ cell exhibits superior capacity retention (against the capacity at the 25^th^ cycle) of 89% compared with that of the cell without mPEG‐P (28%) after 150 cycles at 0.2 A g^−1^.

## 2 Results and Discussion

### 2.1 Noncovalent Interaction Driven Self‐Assembly in mPEG‐P Added Electrolytes

Modified aqueous electrolytes for AZIBs were constructed through mixing water and mPEG‐P at different volume ratios, followed by dissolving a constant concentration of 2 m (m: mol/kg) Zn(OTf)_2_ salt into the mixed solvent. The electrolytes with varied volume fractions of 0%, 0.5%, 1%, and 2% mPEG‐P were adopted to engineer the performance of zinc metal batteries, which are denoted as BE, mPEG‐P0.5, mPEG‐P1, and mPEG‐P2, respectively. The pH and ionic conductivity in various electrolytes were initially investigated (Figure S1, Supporting Information). With the increase in the content of mPEG‐P, the pH of the electrolyte gradually decreases, indicating a gradual increase in proton concentration. It is noteworthy that mPEG‐P1 exhibits the highest ionic conductivity compared to others. The mPEG‐P may ionize partly in water to give 1 or 2 hydrogen ions depending on the pH value of the solution,^[^ ^10^ ^]^ increasing the number of conductive ions. By testing the Zn plating/stripping properties in those electrolytes using Zn//Cu half‐cells (Figure S2, Supporting Information), mPEG‐P1 demonstrated the highest CE and longest cycling stability due to its high conductivity (45.7 ms cm^−2^) and an optimal pH value (4.3, Figure S1, Supporting Information). Besides, to ensure the repeatability and reliability of mPEG‐P additive, the similar performance can be reproduced at identical condition (Figure S3, Supporting Information). Therefore, based on the results, we contrapose physiochemical and electrochemical features of control‐group electrolytes for further investigations, especially for those of mPEG‐P1 which presented the best performance.

**Figure 1:**
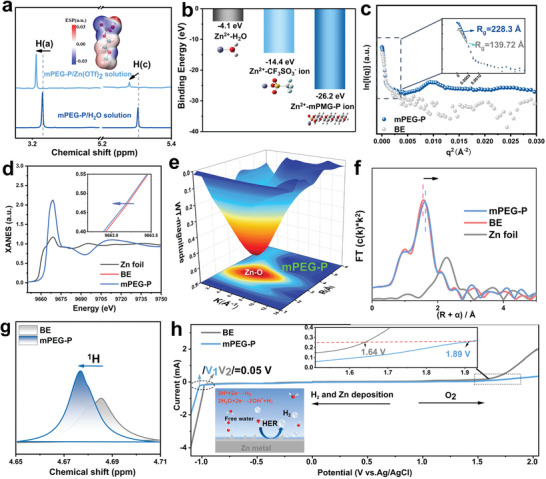
Self‐assembly and properties of electrolytes. a) ^1^H NMR spectra of mPEG‐P/Zn(OTf)_2_ solution and aqueous solution of mPEG‐P (the inset is the electrostatic potential (ESP) plot of mPEG‐P). b) The binding energies of H_2_O, CF_3_SO_3_^−^ ion, and mPEG‐P ion with Zn^2+^ ion. c) The radius of gyration from SAXS experiments for different electrolytes. d) Normalized X‐ray absorption near‐edge structure (XANES) spectra of the Zn foil, Zn(OTf)_2_, and mPEG‐P /Zn(OTf)_2_ electrolytes. e) Wavelet transform images of the EXAFS spectra for mPEG‐P electrolyte. f) Comparison between experimental Zn K‐edge XANES spectra of Zn foil and different electrolytes. g) ^1^H NMR spectra of mPEG‐P/Zn(OTf)_2_ electrolyte and Zn(OTf)_2_ electrolyte. h) Electrochemical stability window of the selected electrolytes testing on Ti electrodes.

Nuclear magnetic resonance (NMR) was used to investigate the molecular interactions in the electrolytes. As shown in Figures S4 and S5 (Supporting Information), the ^1^H in the mPEG‐P molecule mainly has three chemical environments. In the mPEG‐P/Zn(OTf)_2_ solution, all ^1^H peaks of mPEG‐P noticeably shift towards a higher‐field position compared to the aqueous solution of mPEG‐P because the coordination of mPEG‐P with Zn^2+^ and the self‐assembly of mPEG‐P increases the density of the electron cloud around the hydrogen atoms of mPEG‐P (**Figure** **1**a).^[^ ^11^ ^]^ Besides, in contrast to the aqueous solution of mPEG‐P, the ratio of the hydrogen peaks (H_(a):_ H_(c)_) area of the two chemical environments of mPEG‐P in the mPEG‐P/Zn(OTf)_2_ solution undergoes a significant change in proportion, which indicates that the H_(c)_ dissociates from mPEG‐P (Figure 1a; Figure S6, Supporting Information). Moreover, the charge may allow a higher chemisorption activity of mPEG‐P‐derived anions with Zn^2+^. In addition, the electrostatic potential (ESP) plot in Figure 1a indicates that the oxygen‐containing functional groups (*i.e*., C‐O, P = O, P‐OH) exhibit negative ESP values, providing a nucleophilic position for the Zn^2+^, which implies that the oxygen of mPEG‐P has a strong interaction with Zn^2+^.^[^ ^12^ ^]^ Zeta potential measurements of the electrolytes show that the potential value increased from −1.15 mV to −5.20 mV with the addition of mPEG‐P (Figure S7, Supporting Information). This indicates that mPEG‐P‐derived anions can change of electric field in the electrolyte,^[^ ^13^ ^]^ which helps to reduce the coulomb repulsion of Zn^2+^ ions during the deposition process. The interaction of Zn^2+^ with mPEG‐P ions in the electrolyte was further verified by the density functional theory (DFT) simulations (Figure 1b). The coordination ability of water for Zn^2+^ (−4.1 eV) is much smaller than that of mPEG‐P (−26.2 eV), implying that there is a strong binding effect through phosphate groups between Zn^2+^ and mPEG‐P‐derived anions. Therefore, in the Zn(OTf)_2_ solution, mPEG‐P‐derived polyanions undergo electrostatic interactions with Zn^2+^, inducing the formation of the coordination complex.

Previous studies on Zn anodes have proved that hydrophilic functional groups, such as phosphorus‐containing functional groups, can be adsorbed on the metal surface, while the long alkyl chains form a stable molecular film through Van der Waals interactions, enhancing anti‐corrosion performance of the metal interface.^[^ ^10^, ^14^ ^]^ In addition, contact angle experiments further verified that the electrolyte with additives had better wettability with the zinc anodes, promoting the uniformity of Zn^2+^ flow and plating (Figure S8, Supporting Information). Significantly, after 10 min of resting, the contact angle of the mPEG‐P electrolyte on the zinc surface was further reduced due to the aggregation and adsorption properties of the additive on the zinc surface. Small‐angle X‐ray scattering (SAXS) was employed to investigate the aggregation behavior of molecules in electrolytes (Figure 1c; Figure S9, Supporting Information). With the addition of mPEG‐P, the radius of gyration (Rg) of the electrolyte, assessable from the SAXS signal by performing a conventional Guinier analysis,^[^ ^15^ ^]^ increases from 139.72 to 228.3 Å, indicating that mPEG‐P molecules aggregate to form larger clusters.^[^ ^16^ ^]^ Furthermore, the SAXS curve of mPEG‐P/Zn(OTf)_2_ electrolyte aligns well with the (Figure S10, Supporting Information), suggesting that the mPEG‐P molecules self‐assemble into a layered structure.^[^ ^17^ ^]^ Particularly, the mPEG‐P in aqueous electrolyte undergoes the molecular recognition process, in which the polar ether and phosphate groups in the long carbon chains can increase the attraction among molecules through noncovalent interactions such as hydrogen bonding or van der Waals forces, and thus induce the self‐assembly of mPEG‐P. Notably, such aggregates self‐assemble to form organized “hemimicelles” of the mPEG‐P on the Zn metal interface,^[^ ^10^ ^]^ and the phosphate ions of mPEG‐P can interact with the metal surface to form stable ligand covalent bonds.^[^ ^18^ ^]^ To further verify mPEG‐P aggregation model in electrolytes, the simulated structural models were optimized by theoretical simulations, which show different arrangements of mPEG‐P molecules (Figure 1c; Figure S11, Supporting Information). The calculation results show that the total energy of parallel arranged mPEG‐P molecules is about 1.9 eV lower than that of disordered mPEG‐P molecules, indicating that mPEG‐P molecules tend to arrange as heminmicelles in the electrolyte. Moreover, the electrostatic repulsion and steric hindrance between the chain of mPEG‐P also induce them to align vertically near the metal interface. In addition, the chain‐like polyether bonds have better flexibility, allowing mPEG‐P to adapt to the morphology and structure of the metal surface, and increasing the tightness and stability of the hemimicelles.^[^ ^19^ ^]^

X‐ray absorption fine structure analysis (XAFS) was employed to further verify the effects of mPEG‐P additives on Zn^2+^. The Zn K‐edge X‐ray absorption near‐edge structure (XANES) spectra of Zn foils and electrolytes are compared in Figure 1d. The Zn foil displays the smallest near‐edge absorption energy due to its valence state of zero. Compared with the Zn(OTf)_2_ solution, the mPEG‐P/Zn(OTf)_2_ solution exhibits lower energy in the XANE spectrum, proving that mPEG‐P donates more electrons to Zn^2+^.^[^ ^20^ ^]^ Furthermore, the wavelet transformed (WT) EXAFS was conducted to further elucidate the local solvation structure of Zn^2+^ (Figure 1e; Figure S12, Supporting Information). The red region represents the locations where the peaks of R‐space and k‐space coincide, owing to the scattering of Zn‐O.^[^ ^21^ ^]^ Besides, the k^2^‐weighted FT‐EXAFS at Zn K‐edge for Zn(OTf)_2_ electrolytes and mPEG‐P/Zn(OTf)_2_ electrolytes were fitted to analyze the coordination configuration and bond length of Zn^2+^ (Figure 1f, Figures S13, S14 and Table S1, Supporting Information). The fitted coordination number in electrolytes for both structures is ≈6. The Zn─O bond length in Zn(OTf)_2_ electrolytes is ≈2.06 Å. Notably, there are two main Zn‐O scattering paths in mPEG‐P /Zn(OTf)_2_ electrolytes with an average length of ≈1.96 and ≈2.09 Å, which can be assigned to the Zn─O bonds with mPEG‐P additives and H_2_O respectively (Table S1, Supporting Information).

The evolution of hydrogen bonding in various electrolytes was further investigated using Fourier transform infrared (FTIR) spectroscopy (Figure S15, Supporting Information). The O‐H stretching vibration at around 3300 cm^−1^ shifts to a higher wave number after the increase of mPEG‐P, suggesting that the interaction between H_2_O and mPEG‐P weakens the hydrogen bonding network between H_2_O molecules.^[^ ^22^ ^]^ In addition, it is observed from the NMR hydrogen spectrum that the ^1^H signal shifts toward the higher field after the addition of mPEG‐P compared to that of the Zn(OTf)_2_ solution (Figure 1g), indicating a significant enhancement of proton de‐shielding effect and the establishment of hydrogen bonding between mPEG‐P and water molecules.^[^ ^23^ ^]^ The electrochemical stability window (ESW) of the electrolyte was investigated using linear scanning voltammetry (Figure 1h). In comparison to the pure Zn(OTf)_2_ electrolyte, the onset potential of the hydrogen evolution reaction (HER) is suppressed (0.05 V) in the electrolyte with mPEG‐P additive. Similarly, the onset potential of the oxygen evolution reaction (OER) also undergoes a significant delay. The corrosion rate of Zn anodes in different electrolytes was examined by linear polarization test (Figure S16, Supporting Information). The results show that the corrosion current density of zinc anode in mPEG‐P/Zn(OTf)_2_ electrolyte is 0.28 mA cm^−2^, which is much smaller than that in pure Zn(OTf)_2_ electrolyte (3.95 mA cm^−2^). These results confirm that mPEG‐P additive has an anti‐corrosion effect on the zinc anode. FTIR spectroscopy and Raman spectroscopy were used to confirm the adsorption of mPEG‐P on Zn anodes (Figures S17, S18, Supporting Information). The presence of mPEG‐P‐specific C‐H vibrational peaks and P‐O vibrational peaks on the post‐soaked Zn foil are proven. Furthermore, X‐ray photoelectron spectroscopy (XPS) analysis was performed on Zn anodes which were soaked in the mPEG‐P/Zn(OTf)_2_ electrolyte for 4 hours. As shown in Figures S19, S20 (Supporting Information), the characteristic peaks ZFHreferring to mPEG‐P (i.e., C‐O and C‐P) and the phosphate groups are observed in core‐level spectra of C 1*s* and P 2*p*, respectively,^[^ ^18b^ ^]^ indicating the spontaneous adsorption of mPEG‐P on the Zn surface.

### 2.2 Anti‐Calendar Aging of Zn Electrode under Resting Condition

**Figure 2:**
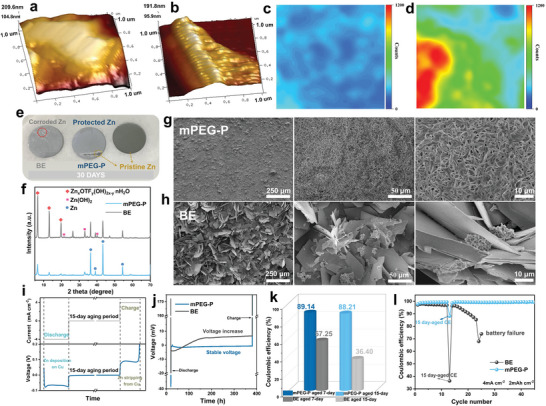
Morphology and anti‐calendar aging performance of Zn electrodes. AFM images and 2D Raman mapping on the surfaces of metallic zinc electrodes after soaking in electrolytes with mPEG‐P a, c) and without mPEG‐P b, d) for 7 days. e Optical images, f) XRD patterns, and g, h) SEM images of Zn foils after soaking in different electrolytes for 30 days. i) Electrochemical test procedure for quantifying aging‐induced capacity loss for Zn//Cu cells. j) Voltage changes of Zn//Cu cells during resting in 15 days. k) The CE_aged_ of Zn//Cu cells aged for 7 days and 15 days using different electrolytes, respectively. l) CE of Zn//Cu cells using electrolytes with/without mPEG‐P including the 15‐day aging procedure.

With the steadily growing amount of globally installed stationary battery energy storage systems, aging aware operation of these systems becomes increasingly important.^[^ ^24^ ^]^ However, to the best of our knowledge, there are few studies have been conducted on electrode corrosion during calendar aging and its effect on battery performance. As mentioned above, the mPEG‐P‐derived polyanions strongly adsorb and self‐assemble on the electrode interface, which is probably to improve the anti‐calendar aging of Zn electrodes. Therefore, the corrosion behavior during aging process was initially investigated through immersion experiments using Zn metal. In Figure S21 (Supporting Information), following 4 hours of immersion in the pure Zn(OTf)_2_ electrolyte, the scanning electron microscopy (SEM) images reveal the presence of uneven flakes on the electrode surface. In contrast, the surface of the Zn foil remains flat. Subsequently, after 7 days of immersion in the respective electrolytes, the morphological analysis of the Zn foil surface was conducted using atomic force microscopy (AFM) (**Figure** **2**a,b). The results demonstrate the formation of sharp protrusions on the surface of the Zn foil immersed in pure Zn(OTf)_2_ electrolyte. As expected in Figure 2c,d, by conducting 2D Raman mapping analysis at 1035.15 cm^−1^ on the soaked Zn foil surface, it is found that the surface of Zn foil in the Zn(OTf)_2_ electrolyte exhibits zinc hydroxide sulfonate by‐products, which is consistent with the findings of the previous study.^[^ ^25^ ^]^ Moreover, the intensity of these by‐products on the surface of Zn foil in the mPEG‐P/Zn(OTf)_2_ electrolyte is significantly lower. After immersing the Zn foil in the Zn(OTf)_2_ electrolyte for 30 days, notable corrosion changes are observed on its surface (Figure 2e). Massive by‐products are formed, leading to localized protrusions and corrosion craters. However, it is essential to emphasize that the addition of mPEG‐P resulted in the formation of a uniform greyish‐white protective film on the surface of the Zn foil. Furthermore, we subsequently used tweezers to disrupt the protective layer on the zinc foil surface (Figure S22, Supporting Information). Upon removal of the protective layer, the zinc foil exhibited the same color characteristics as the original zinc foil. Besides, consistent results were also observed by utilizing industrial optical microscopy to examine the Zn foils after immersion (Figure S23, Supporting Information).

To determine the crystal phases of the Zn foil surface, X‐ray diffraction (XRD) was performed on the Zn foil after 30 days of immersion (Figure 2f). The sample soaked in pure Zn(OTf)_2_ electrolyte exhibits the presence of Zn(OH)_2_ and Zn_x_OTf_y_(OH)_2x‐y_·nH_2_O (ZFH) on the surface, while the Zn foil immersed in the mPEG‐P/Zn(OTf)_2_ electrolyte exhibits only a small amount of ZFH. The formation process of ZFH is similar to that of ZnSO_4_(OH)_6_·5H_2_O.^[^ ^26^ ^]^ The proton‐involved reaction leads to an increase in the local OH^−^ concentration neighboring the electrode surface, which produces Zn(OH)_2_ first and reacts with triflate to generate Zn_x_OTf_y_(OH)_2x‐y_·nH_2_O subsequently. It is seen that the mPEG‐P additive can effectively inhibit the continuous corrosion reaction on the surface of Zn foil. In addition, the microscopic morphology of Zn foils immersed in different electrolytes for 30 days was further investigated by scanning electron microscopy (SEM) (Figure 2g,h). After immersion in the Zn(OTf)_2_ electrolyte, dramatic flaky by‐products are observed on the surface of Zn foils. These by‐products accumulate haphazardly on the surface, displaying random orientation and uneven distribution. This loose layer cannot effectively block the electrolyte from reaching the fresh Zn surface, that is, the corrosion reactions on the Zn electrode is hard to be terminated through the inconsecutive passivation layer.^[^ ^27^ ^]^ In contrast, there is a relatively flat and felt‐like microstructure existing on the surface of the Zn foil observed from the sample immersed in the mPEG‐P/Zn(OTf)_2_ electrolyte. In addition, the densely felty passivation layer contains horizontally deposited ZFH, which has high Zn^2+^ conductivity and low electronic conductivity.^[^ ^28^ ^]^ The formation of the passivation layer guides dendrite‐free Zn plating underneath the protective layer rather than on the layer surface, thereby avoiding electrolyte‐induced side reactions.^[^ ^4c^ ^]^ Besides, the XRD pattern of the zinc foil that had been soaked in the mPEG‐P aqueous solution for 7 days did not show the presence of diffraction peaks of the by‐products on the surface, which suggests that the additives do not cause corrosion of the zinc foil (Figure S24, Supporting Information).

Measurement of the CE of Zn//Cu half‐cells is used to evaluate the performance of Zn plating/stripping. However, under the circumstances of practical applications and experimental measurements, AZIBs have to experience varied degrees of the calendar aging period before (dis)charge process, which is usually neglected in previous studies. Therefore, to investigate the effect of the aging process on Zn plating/stripping, a long‐resting procedure setting (7 and 15 days) was carried out for the cells in operation. This allows us to gain more deep insights into the aging behavior of Zn anodes with electrochemical performance. Specifically, the Zn//Cu cells have a discharge process to deposit 2 mAh cm^−2^ of Zn (Q_P_) on the Cu electrode at a current density of 4 mA cm^−2^, and then the cells are aged at room temperature for either 7 or 15 days (Figure 2i). After the aging process, a charging process is initiated, during which the Zn on the copper electrode undergoes stripping (Q_s_). The CE of this aging process can be defined as CE_aged_:

(1) $$CE_{\mathrm{aged}}=\frac{\mathrm{Stripping}\;\mathrm{capacity}\;\left(Qs\right)}{\mathrm{Plating}\;\mathrm{capacity}\;\left(Qp\right)}\;\times 100\%$$

As illustrated in Figure 2j and Figure S25 (Supporting Information), the voltage of the Zn//Cu cells with Zn(OTf)_2_ electrolyte exhibits a significant increase during the aging period of 7 and 15 days. Notably, the voltage of the Zn//Cu cell with mPEG‐P electrolyte remains consistently stable, indicating that mPEG‐P additives effectively inhibit the continuous growth of by‐products on the anode surface. The same conclusion can also be verified from the SEM morphology of the anode in Zn//Cu batteries after 7days‐resting time (Figure S26, Supporting Information). In Figure 2k and Figure S27 (Supporting Information), after 7 days of aging, the Zn//Cu cell with Zn(OTf)_2_ electrolyte exhibits Q_s_ of 0.728 mAh cm^−2^ and CE_aged_ of 57.25%. However, the cell with mPEG‐P additive shows superior plating/stripping performance reflecting the Q_s_ of 1.784 mAh cm^−2^ and a CE_aged_ of 89.14%. Besides, the CE_aged_ of the Zn//Cu cell with Zn(OTf)_2_ electrolyte is only 36.40% after a 15‐day aging treatment, while the mPEG‐P‐added battery maintains a superb level of CE_aged_ at 88.21%. In the Zn(OTf)_2_ electrolyte, the side reaction of zinc with the electrolyte produces by‐products such as Zn(OH)_2_ and ZFH, and this irreversible depletion of zinc causes capacity decay. During operation after 15 days of aging treatment, the CE of the cell with Zn(OTf)_2_ electrolyte decreases rapidly and fails in the subsequent 12 cycles, and finally deteriorates the cycling performance. In contrast, the Zn//Cu cell with mPEG‐P presents an average CE over 99% along with high stability in the subsequent 30 cycles (Figure 2l). Hence, the in situ protective layer formed in the aging process significantly suppresses the static corrosion and has excellent plating/stripping performance of zinc ions, improving the cycling stability of AZIBs.

### 2.3 Enhanced Mass Transfer Kinetics and In Situ SEI Formation on Zn Anodes

**Figure 3:**
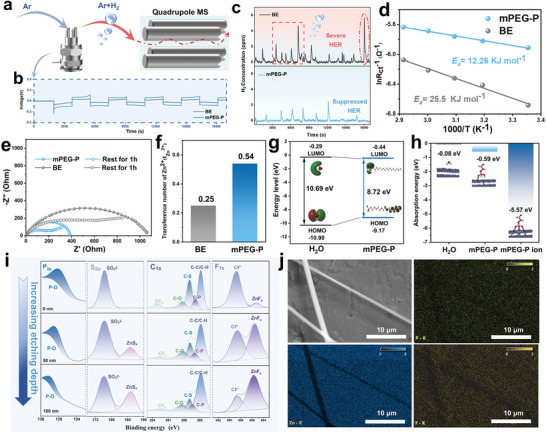
Enhanced mass transfer kinetics of zinc ion and in situ SEI formation on Zn anodes. a) in situ DEMS measurements in the Swagelok cell and b) the corresponding voltage profiles at 3 mA cm^−2^. c) H_2_ evolution during in situ DEMS measurements. d) Arrhenius curves and comparison of activation energies. e) EIS spectra of the Zn//Zn symmetric cells in electrolytes with/without mPEG‐P additive before and after resting for 1 hour. f) Zn^2+^ transference number (t_Zn_^2+^) comparison of two electrolyte systems. g) The calculated HOMO‐LUMO gap of H_2_O and mPEG‐P molecule. h) The calculated adsorption energy of H_2_O, mPEG‐P molecule, and mPEG‐P ion on the Zn (002) surface. i) The core‐level XPS spectra with sputtering depth analysis for the electrode after cycling for 50 hours at 1 mA cm^−2^ in the mPEG‐P/Zn(OTf)_2_ electrolyte. j) SEM image and EDS elemental mapping of the Zn anodes after cycling for 20 hours at 1 mA cm^−2^.

To further explore the interfacial dynamics of Zn anodes with mPEG‐P additive, electric double layer capacitance (EDLC) measurements of Zn anodes were carried out in different electrolytes (Figures S28, S29, Supporting Information). It displays a lower EDLC in mPEG‐P/Zn(OTf)_2_ electrolyte (31.23 µF cm^−2^) than in pure Zn(OTf)_2_ electrolyte (46.52 µF cm^−2^). This is due to the self‐assembly and adsorption of mPEG‐P at the interface, which enriched the electric double‐layer (EDL), thereby increasing steric hindrance.^[^ ^29^ ^]^ Besides, to further investigate the influence of interfacial adsorption characteristics on the side reaction, we examined the generation of hydrogen during zinc plating/striping process by in situ electrochemical differential mass spectrometry (DEMS) for Zn//Zn symmetric cells with different electrolytes. These batteries were assembled at identical conditions and operated at 3 mA cm^−2^ (**Figure** **3**a). Figure 3b shows the voltage‐time curve of the in situ symmetric cell during cycling. As shown in Figure 3c, the cell with the Zn(OTf)_2_ electrolyte vigorously generated hydrogen during cycling, as evidenced by the significantly higher H_2_ concentrations along with the occurrence of high frequency signals. In contrast, the cell with the mPEG‐P/Zn(OTf)_2_ electrolyte shows fewer amount of hydrogen production along with low‐frequency generation. This results successfully demonstrate the effectiveness of HER suppression through the aid of mPEG‐P additive, which can contribute to the safety and stability of zinc‐based energy storage devices.

Since the desolvation of Zn^2+^ is the main rate‐determine step for ion migration, the activation energy (*E_a_*) was evaluated by the Arrhenius equation to study Zn^2+^ mass transfer kinetics. *E_a_* was obtained by electrochemical impedance spectroscopy (EIS) test at 25–70°C (Figures S30, S31, and Table S2, Supporting Information). The Zn electrode in mPEG‐P/Zn(OTf)_2_ electrolyte shows a lower activation energy (12.26 kJ mol^−1^) than that in pure Zn(OTf)_2_ electrolyte (25.5 kJ mol^−1^) (Figure 3d), indicating an improved desolvation capability along with minimized parasitic reactions caused by water at the interface.^[^ ^30^ ^]^ Meanwhile, lower zinc ion mobility may lead to a high Zn‐ions concentration gradient on the surface of the electrode, which generates a strong interfacial electric field and exacerbates the growth of harmful dendrites.^[^ ^31^ ^]^ As shown in Figure 3e, compared with pure Zn(OTf)_2_ electrolyte, the Zn//Zn battery with mPEG‐P electrolyte exhibits lower charge‐transfer resistance. Besides, even after standing for an hour, the charge‐transfer resistance is much smaller than that of pure Zn(OTf)_2_ electrolyte. The transfer number of Zn^2+^ (t_Zn_^2+^), which quantifies the relative ionic diffusivity in the electrolyte, was determined through experimental measurements (Figures S32, S33, Supporting Information). In Figure 3f, the Zn^2+^ transfer number in the Zn//Zn symmetric cell with Zn(OTf)_2_ electrolyte is only 0.25. Notably, the Zn^2+^ transfer number significantly increases to 0.54 in the cell with mPEG‐P additives. Moreover, the high electronegativity of the oxygen atoms in the ether group creates a localized region of negative charges, which helps to directionally induce the Zn^2+^ to migrate quickly and uniformly toward the Zn electrode, reducing concentration polarization and enhancing rate performance.^[^ ^2a^ ^]^ The surface functional groups of the cycled Zn anode were tested by FTIR, as shown in Figures S34 and S35 (Supporting Information). The mPEG‐P characteristic peaks are observed at the cycled Zn anode, and it is worth noting that the P‐O vibrational peaks and P‐O‐C vibrational peak have an obvious red‐shift, which is due to the combination of mPEG‐P‐derived anions with the surface of the Zn anode to form a ligand covalent bond (P‐O‐Zn).^[^ ^32^ ^]^ Figure 3g summarizes the energy levels of the lowest unoccupied molecular orbital (LUMO) and highest occupied molecular orbital (HOMO) for H_2_O and mPEG‐P. According to molecular orbital theory, the narrower the bandgap between the LUMO and HOMO energy levels, the stronger the electron transfer ability, which is beneficial for the adsorption of solvent molecules on Zn.^[^ ^33^ ^]^ The simulated bandgaps of H_2_O and mPEG‐P are 10.69 eV and 8.72 eV respectively, indicating that mPEG‐P is more inclined to adsorb on Zn than H_2_O. Furthermore, based on DFT simulations, the adsorption energies of mPEG‐P and mPEG‐P polyanions on the Zn (002) crystal plane are −0.59 eV and −5.57 eV respectively, while the adsorption energy of H_2_O molecule is only −0.08 eV. It confirms stronger adsorption between the additive and zinc metal (Figure 3h). This can help the establishment of the H_2_O‐deficient Zn‐mPEG‐P molecular interface, hence reducing the interfacial side reactions.^[^ ^34^ ^]^

To elucidate the specific composition of the SEI layer, XPS with different depths etched by continuous argon (Ar^+^) etching was adapted to characterize cycled Zn anodes (Figure 3i). In the core‐level spectra of P 2*p*, the signal intensity of P decreases with increasing sputtering depth. In the core‐level spectra of S 2*p* and F 1*s*, the signals of inorganic components ZnF_x_ (685.7 eV) and ZnS_x_ (163.2 eV) are significantly enhanced with increasing sputtering depth, and the signals of S‐O and C‐F, originating from the decomposition of triflate anion, exhibit a decrease in intensity. In the core‐level spectra of C 1*s*, C‐S originates from the decomposition of triflate anion, while C‐O and C‐P are the characteristic functional groups of mPEG‐P. The high rigidity of the inorganic component (ZnF_x_/ZnS_x_) significantly inhibits the growth of zinc dendrites, while the high toughness of the organic component (i.e., C‐O, C‐P, P‐O) of mPEG‐P ensures that the SEI layer is sufficiently flexible. Both of them ensure that the SEI layer is more reliable and sustainable than those containing only inorganic or organic components.^[^ ^33^ ^]^ From the energy dispersive X‐ray spectroscopy (EDS) of the Zn anode after cycling for 20 hours in Figure 3j, there is a uniform distribution of Zn and P which were detected through element mappings, suggesting the homogeneous coverage of mPEG‐P molecules on the Zn anode and the Zn‐mPEG‐P molecular interfacial layer is constructed on the surface. Moreover, after 110 hours of cycling, the EDS of the Zn anode exhibits the presence of P, indicating excellent stability of the interfacial layer during the cycling process (Figure S36, Supporting Information). Subsequently, the cross‐sectional images of the Zn anode after cycling for 20 hours show the presence of the protective SEI layer on the surface without clusters of by‐products (Figure S37, Supporting Information).

### 2.4 Improvement of Zn Deposition in Two‐Phase Interface by Engineered In Situ SEI

**Figure 4:**
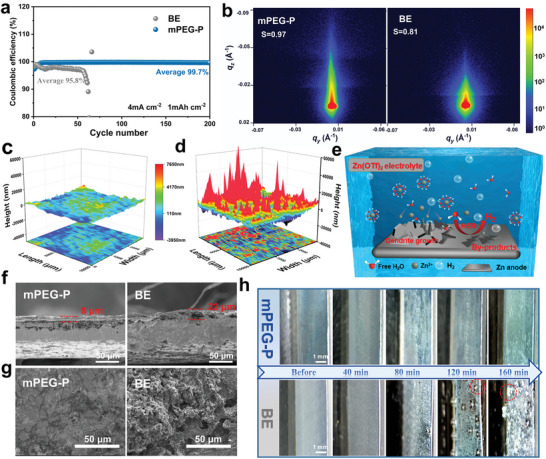
Zn deposition behavior in different electrolytes. a) CE and cycling performance of Zn//Cu cells under current density of 4 mA cm^−2^ with 1 mAh cm^−2^. b) 2D GISAXS data at the cycled Zn anodes in mPEG‐P. and BE (cycling for 5 h at 1 mA cm^−2^). 3D surface‐profilometry images of cycled Zn anodes in different electrolytes with mPEG‐P c) or BE d), respectively. e) The schematic diagrams of Zn deposition behaviors in Zn(OTf)_2_ electrolyte. f) The cross‐sectional images of Zn anodes after 50 cycles in electrolytes with/without mPEG‐P. g) The SEM images of Zn anodes in electrolytes with/without mPEG‐P after cycling for 50 h at 2 mA cm^−2^. h) in situ optical microscope images of the Zn anodes cycled in Zn(OTf)_2_ electrolyte and mPEG‐P/Zn(OTf)_2_ electrolyte using the industrial optical microscope.

To further reveal the modulated Zn growth behavior enabled by the hybrid SEI layer, the CE of Zn plating/stripping was characterized by Zn//Cu half‐cells. As shown in **Figures** **4**a and S38 (Supporting Information), the Zn//Cu cell with mPEG‐P/Zn(OTf)_2_ electrolyte maintains a high average CE of 99.7% over 200 cycles at 4 mA cm^−2^ and 1 mAh cm^−2^, whereas the cell with pure Zn(OTf)_2_ electrolyte has poor cycling stability (CE = 95.8%) with severe fluctuations and relatively faster failure. Furthermore, the reproduced CE performance of Zn//Cu cells also demonstrates the reliability of the effective role of additive (Figure S39, Supporting Information). Besides, in the Zn(OTf)_2_ electrolyte, the voltage gap exhibits a progressive increase of 40 mV between the 30^th^ and 50^th^ cycles, while the change of voltage gap in the mPEG‐P/Zn(OTf)_2_ electrolyte was merely 2.3 mV, implying a significant reduction in energy loss during cycling (Figure S40, Supporting Information). According to the classical nucleation theory,^[^ ^35^ ^]^

(2) $$\Delta G_{crit}=\frac{{\left(\nu -1\right)}^{\nu -1}}{\nu^{\nu }}\;\frac{\varphi^{\nu }\alpha_{\nu D}^{\nu }}{{\left(ze\left|\eta \right|\right)}^{\nu -1}}$$

(3) $$\ln J\;=\;\ln A_{J}-\frac{{\left(\nu -1\right)}^{\nu -1}}{\nu^{\nu }}\frac{\varphi^{\nu }\alpha_{\nu D}^{\nu }}{kT{\left(ze\left|\eta \right|\right)}^{\nu -1}}$$

It is noteworthy that the above formulas are applicable in both 2D and 3D nucleation models, with only an adjustment required for the value of *ν*. *ν* is the dimensionality of the cluster: *ν* = 1, 2, or 3. *A_J_* is a constant of proportionality, *k* is the boltzmann constant, *T* is the thermodynamic temperature, *z* is the number of electrons, *φ* is the specific boundary energy, and *α* is the constant of proportionality, which is related to the dimensionality of diffusion. Therefore, according to equations 2 and 3, the increase of |*η*| (50 mV) in mPEG‐P/Zn(OTf)_2_ electrolyte, as tested through Cyclic Voltammetry (CV) (Figure S41, Supporting Information), results in a decrease in *ΔG_crit_* and an increase in *J*, which help to promote the formation of fine crystalline zinc deposits and avoid the accumulation of initial protrusions.^[^ ^35b^ ^]^ In addition, the nucleation and growth process of metallic zinc on the electrode surface can be further tested using the chronoamperometry (CA) method (Figure S42, Supporting Information). In the pure Zn(OTf)_2_ electrolyte, the current increases continuously due to non‐uniform nucleation caused by 2D uncontrolled diffusion of Zn^2+^ and the tip effect. Adding mPEG‐P stabilizes the current more quickly by promoting three‐dimensional diffusion behavior dominant in Zn^2+^ nucleation, resulting in uniform deposition on the surface.

Grazing incidence small‐angle X‐ray scattering (GISAXS) was applied to shed light on the molecular arrangement of the surface layer. As shown in Figure 4b and Figure S43 (Supporting Information), the zinc anode surface exhibits a higher Herman's orientational order parameter (s) after cycling in the mPEG‐P/Zn(OTf)_2_ electrolyte, indicating a more ordered surface structure which facilitates the formation of optimized ion conduction pathways and stable electrochemical reaction processes.^[^ ^36^ ^]^ The 3D surface profilometry images also show the surface morphology of the Zn anode after cycling for 20 cycles over a large area (Figure 4c,d). In the Zn(OTf)_2_ electrolyte, the Zn anode exhibits a greater surface roughness, with a maximum height exceeding 6,000 nm. In sharp contrast, the Zn anode shows a much flatter surface after cycling in the mPEG‐P/Zn(OTf)_2_ electrolyte. Notably, this disordered buildup of flaky by‐products on the electrode hampers ion transport.^[^ ^37^ ^]^ In addition, hydrogen generated by the corrosion reaction gradually accumulates on the surface and isolates part of the Zn metal from the electrolyte, restraining the area from participating in redox reactions (Figure 4e).^[^ ^38^ ^]^ In addition, the cross‐sectional image of the Zn anode in the mPEG‐P/Zn(OTf)_2_ electrolyte after 50 cycles shows a dense and uniform Zn deposition layer of 8 µm thickness, whereas the cross‐section of the Zn anode in the pure Zn(OTf)_2_ electrolyte is unevenly deposited, reaching 22 µm (Figure 4f). The morphological changes of the zinc anode during the cycling process were observed by scanning electron microscopy (SEM) (Figure 4g). After 50 hours of cycling in the mPEG‐P/Zn(OTf)_2_ electrolyte, the Zn anode shows a flatter and dendrite‐free SEI surface. While blocky cluster protrusions with irregular shape were observed in pure Zn(OTf)_2_ electrolyte. A similar phenomenon was observed in the SEM images after 100 h of cycling (Figure S44, Supporting Information). X‐ray diffraction (XRD) of the zinc foils after 50 hours of cycling in the mPEG‐P/Zn(OTf)_2_ electrolyte shows that only peaks corresponding to metallic zinc are detected on the zinc anode, whereas peaks corresponding to the ZFH are observed in the zinc anode of the zinc circulating in the Zn(OTf)_2_ electrolyte (Figure S45, Supporting Information). This indicates that the addition of mPEG‐P can inhibit the deleterious side reactions between metallic zinc and the electrolyte. Furthermore, an in situ optical electrochemical cell (Zn//Zn symmetric cell) was fabricated to observe the surface morphology of the Zn anodes during cycling using an industrial optical microscope (Figure 4h, Figure S46, Supporting Information). Compared to previous studies, we adopt a relatively larger observation scale to reduce the randomness and limitations of localized microscopic observations on the surface of the Zn anodes. At a current density of 3 mA cm^−2^, a large number of bubbles and by‐products are observed on the surface after cycling in pure Zn(OTf)_2_ electrolyte, posing a high risk of short circuit. However, using the electrolyte with mPEG‐P, the surface remains uniformly stable throughout the cycling process. Additionally, the surface morphology of the Zn anodes was then examined by SEM after 80 minutes of cycling in the in situ optical electrochemical cell (Figure S47, Supporting Information). In the Zn(OTf)_2_ electrolyte, an unevenly deposited mass forms on the surface of the Zn anode. In contrast, the surface of the Zn anode is more uniform and dense after cycling in the mPEG‐P/Zn(OTf)_2_ electrolyte.

### 2.5 Electrochemical Performance of Zn Anodes

**Figure 5:**
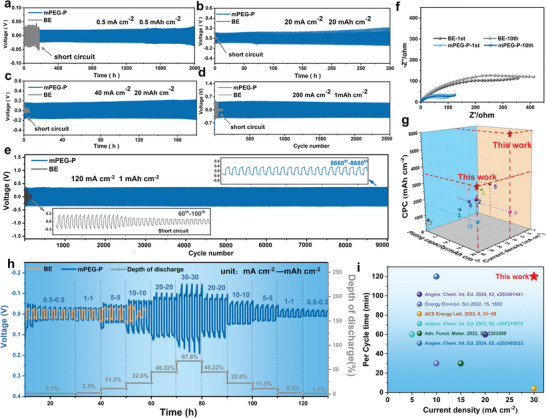
Electrochemical stability of Zn anodes in different electrolytes. Long‐term galvanostatic cycling performance of Zn//Zn symmetric batteries in electrolytes with/without mPEG‐P additive: a) 0.5 mA cm^−2^ and 0.5 mAh cm^−2^. b) 20 mA cm^−2^ and 20 mAh cm^−2^. c) 40 mA cm^−2^ and 20 mAh cm^−2^. d) 200 mA cm^−2^ and 1 mAh cm^−2^. e) 120 mA cm^−2^ and 1 mAh cm^−2^. f) EIS spectra of the symmetric cells in electrolytes with/without mPEG‐P additive after the first cycle and the tenth cycle. g) Comparison of the CPC of this work with other reported literature. h) Rate performance of symmetric cells in electrolytes with/without mPEG‐P at the current densities and plating capacity from 0.5 mA cm^−2^‐0.5 mAh cm^−2^ to 30 mA cm^−2^‐30 mAh cm^−2^. i) The comparison of current density and per cycle time in rate performance with reported literature.

Operation with stability and long lifespan at high current density and high capacity is a key requirement for achieving high‐performance AZIBs. The enhancement of electrochemical stability by mPEG‐P additive induced in situ SEI was further investigated through Zn//Zn symmetric cells (**Figure** **5**a–e). Under a low current density of 0.5 mA cm^−2^, the cells with the mPEG‐P additive exhibit excellent stability with a cycling time longer than 2000 h, while the cell in Zn(OTf)_2_ electrolyte has a poorer cycling stability of only 150 h. Additionally, it is seen that the Zn//Zn symmetric batteries with mPEG‐P exhibit superior performance under ultrahigh plating capacity compared with reported works, those are, the long cycles for 300 h at 20 mA cm^−2^ (20 mAh cm^−2^) and 180 h at 40 mA cm^−2^ (20 mAh cm^−2^), while the symmetric cell with Zn(OTf)_2_ electrolyte experiences an instantaneous short circuit. Moreover, to verify the reliability of mPEG‐P additives, the similar performance can be reproduced at identical condition (Figures S48, S49, Supporting Information). Impressively, when the current density further increases to 200 mA cm^−2^, a highly stable Zn plating/stripping process can be still achieved for nearly 2500 cycles, clearly demonstrating the effectiveness of mPEG‐P for ultrafast Zn^2+^ mass transfer rate. Moreover, the cells with the mPEG‐P additive exhibit excellent stability over 9000 cycles at 120 mA cm^−2^, while the symmetric cell with Zn(OTf)_2_ electrolyte only operates for 70 cycles. Furthermore, the symmetric cell in mPEG‐P/Zn(OTf)_2_ electrolyte exhibits lower charge transfer resistance for both the 1^st^ and 10^th^ cycle compared to the Zn(OTf)_2_ electrolyte, indicating that the electrode interface of the cell with mPEG‐P additives has better charage conduction kinetics (Figure 5f). Additionally, Figure 5g illustrates a comparison of the cumulative plated capacity (CPC) values achieved at different current densities and plating capacities with values reported in other works (Table S3, Supporting Information). It is worth noting that the Zn//Zn symmetric batteries with mPEG‐P additive achieve ultrahigh CPC values of 6 Ah cm^−2^ and 3.6 Ah cm^−2^ at current densities of 20 and 40 mA cm^−2^, respectively.

The rate performance of batteries was investigated at different current densities ranging from 0.5 to 30 mA cm^−2^, with the depth of discharge (DOD) ranging from 1.1% to 67.8% (Figure 5h). It is worth noting that, unlike the commonly used plating capacity (1 mAh cm^−2^) in gradient rate tests observed from previous studies, the symmetric cells with mPEG‐P additive can withstand a record‐high plating capacity of 30 mAh cm^−^^2^ in room temperature, while the symmetric cell of pure Zn(OTf)_2_ shows a short circuit at 10 mAh cm^−2^. Moreover, the DOD of the zinc anode reached 67.8% under the testing condition of 30 mA cm^−2^ and 30 mAh cm^−2^. This implies that the batteries with mPEG‐P additive possess high energy efficiency, thus providing longer usage time and wide scope of applications in stationary battery energy storage systems. In addition, the testing parameters (plating capacity and current density) for the rate capability of the symmetric batteries with mPEG‐P are compared with the previously reported works, demonstrating the outstanding rate performance in this field (Figure 5i).

### 2.6 Electrochemical Performance of Full Cells

**Figure 6:**
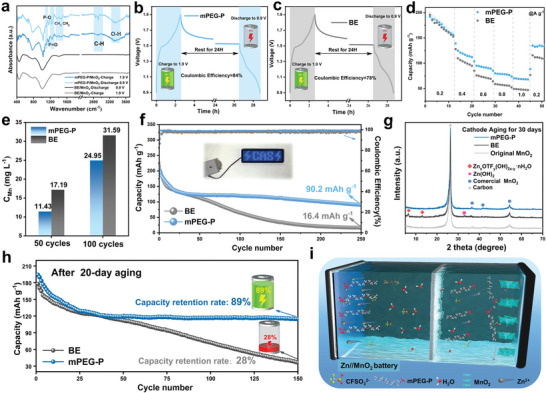
Full cell performance evaluation. a) Ex situ FTIR spectra of the MnO_2_ cathodes in Zn(OTf)_2_ electrolyte and mPEG‐P/Zn(OTf)_2_. Self‐discharge curves of full cells in electrolytes b) without and c) with mPEG‐P additive. d) Comparison of capacities of full cells at various current densities. e) The concentration of Mn in different electrolytes after 50 and 100 cycles at a current density of 0.3 A g^−1^. f) Cycling performance at a current density of 0.2 A g^−1^ (the inset is the LED device powered by two coin cells). g) XRD patterns of MnO_2_ cathodes after aging for 30 days. h) Cycling performance at a current density of 0.2 A g^−1^ after 20‐day aging process. i) The proposed mechanism for mPEG‐P additive in the full cell.

The Zn//MnO_2_ full cells were assembled using commercial MnO_2_ powder as a cathode material to investigate the effect of mPEG‐P on the stability of the cathode material. The crystallographic characterization of the commercial MnO_2_ powder is shown in Figure S50 (Supporting Information). As shown in Figure S51 (Supporting Information), the specific current density of the redox peaks with the mPEG‐P additive is higher, suggesting that mPEG‐P may stabilize MnO_2_, achieving a higher current density response. FTIR was performed on the cycled MnO_2_ cathode (**Figure** **6**a). The infrared vibration peaks corresponding to mPEG‐P are observed on the surface of the MnO_2_ cathode under fully (dis)charge states, indicating that mPEG‐P can adsorb onto the cathode surface through noncovalent interactions. Significantly, the adsorption of mPEG‐P on the surface of MnO_2_ can decrease the direct contact and reaction between active water molecules and manganese dioxide.^[^ ^39^ ^]^ This weakens the bond between MnO_2_ cathodes with water and limits the dispersion of H_2_O, altering the surface characteristics of the MnO_2_ cathode, thereby helping to inhibit the dissolution of MnO_2_ cathodes.^[^ ^40^ ^]^ To further evaluate the interfacial stability of the batteries, we monitored the self‐discharge behavior of the batteries through 24 hours of resting after the charging procedure (Figure 6b,c). The CE of the battery with mPEG‐P/Zn(OTf)_2_ electrolyte reaches up to 84%, while the CE of the battery with Zn(OTf)_2_ electrolyte is only 78%. This verified the ability of mPEG‐P to suppress the side reactions, resulting in a robust electrode surface. Moreover, the full cells exhibit excellent rate performance in the current density range of 0.1 to 1 A g^−1^ (Figure 6d). To further certify the role of mPEG‐P in enhancing the stability of the cathode materials, the inductively coupled plasma optical emission spectrometer (ICP‐OES) was conducted to accurately measure the concentration of dissolved Mn ions within different electrolytes after cycling tests (Figure 6e). Compared to the Zn(OTf)_2_ electrolyte, the mPEG‐P/Zn(OTf)_2_ electrolyte shows a significant decrease in dissolved Mn after both 50 and 100 cycles. Benefiting from the addition of mPEG‐P, the Zn//MnO_2_ cell with mPEG‐P achieves stable cycling with the capacity of 90.2 mAh g^−1^ after 250 cycles at a current density of 0.2 A g^−1^, while the capacity of the cell without mPEG‐P is only 16.4 mAh g^−1^ (Figure 6f). In addition, it is seen that the two as‐prepared coin cells in series can power a light‐emitting diode (LED), thus validating the practical utility of the mPEG‐P additive in AZIBs applications.

To verify the enhancement of mPEG‐P on the calendar life and anti‐aging performance of batteries, full‐cell aging tests were also conducted. After resting for 30 days in different electrolytes, XRD was performed on the MnO_2_ cathode (Figure 6g). The diffraction peaks referring to the Zn(OH)_2_ and ZFH are detected on the cathode of the cell with Zn(OTf)_2_ electrolyte. In contrast, there are no miscellaneous phases existing on the cathode with mPEG‐P/Zn(OTf)_2_ electrolyte. It is seen that the mPEG‐P can effectively inhibit the accumulation of by‐products on the cathode. Furthermore, after 20 days of aging, the full cells undergo constant current (dis)charge measurements to verify the distinguished performance in different electrolytes. As shown in Figure 6h, the cell using mPEG‐P/Zn(OTf)_2_ electrolyte maintains a capacity retention rate of 89% after 150 cycles at 0.2 A g^−1^ against the capacity at the 25^th^ cycle, while the battery in Zn(OTf)_2_ electrolyte has a capacity retention rate of only 28%. Moreover, to ensure the repeatability and reliability of mPEG‐P additive, the similar performance of full cells can be reproduced at identical condition (Figures S52–S54, Supporting Information). Therefore, the mPEG‐P additive provides effective protection against the dissolution of cathode materials and exhibits significant importance in realizing the high reversible capacity (Figure 6i). In addition, to verify the wide‐ranging effect of additives on cathode materials, the vanadium‐based full‐cell tests were tested. NH_4_V_4_O_10_ (NVO) was synthesized as cathode material. The crystallographic characterization of the synthesized NVO powder is shown in Figure S55 (Supporting Information). The self‐discharge behavior of the Zn//NVO batteries was monitored through 24 hours of rest after a fully charged state to further evaluate the interfacial stability of the batteries (Figure S56, Supporting Information). The CE of the battery with mPEG‐P/Zn(OTf)_2_ electrolyte reaches up to 96.7%, while the CE of the battery with Zn(OTf)_2_ electrolyte is only 92.5%. Benefiting from the addition of mPEG‐P, the Zn//NVO batteries achieve more stable cycling with a capacity of 295.6 mAh g^−1^ after 250 cycles at a current density of 2 A g^−1^, while the capacity of the cell without mPEG‐P is only 15.0 mAh g^−1^ (Figure S57, Supporting Information). Moreover, the charge/discharge curves show the reversibility of the Zn^2+^ intercalation /deintercalation process from the 20th to the 200th cycle, indicating the good compatibility of the mPEG‐P electrolytes and NVO cathode (Figure S58, Supporting Information).

## 3 Conclusion

In summary, the mPEG‐P containing multifunctional polar groups are employed to engineer the in situ flexible SEI to optimize the Zn^2+^ mass transfer in zinc metal batteries. It indicates that the in situ formed SEI can significantly boost the reaction kinetics and tremendously extend the calendar life of the AZIBs. Experimental results and theoretical calculations confirm that mPEG‐P polyanions exhibit strong adsorption activity with the Zn anode. More specifically, the mPEG‐P accumulates through noncovalent interactions, and self‐assembles into vertical hemimicelles, forming channels for the rapid mass transfer of zinc ions through polyethers of mPEG‐P chains. Consequently, the Zn//Zn symmetric batteries exhibit superior lifespan over 9000 cycles at an ultrahigh current density of 120 mA cm^−2^, along with stable cycling performance for 2500 cycles at a record‐high current density of 200 mA cm^−2^, and the symmetric cell can withstand up to an unprecedented plating capacity of 30 mAh cm^−2^ during rate test. Furthermore, our results also validate the regulation in ZFH deposition and modulation of in situ organic‐inorganic gradient SEI formation, which are essential for excellent calendar life and battery performance. Specifically, the CE of the Zn//Cu cell is 88.21% in the first cycle after 15 days of calendar aging treatment, which is much higher than the mPEG‐P‐free cell (36.4%). Remarkably, after 20‐day aging, the capacity retention rate of Zn//commercial MnO_2_ cell is 89% after 150 cycles under 0.2 A g^−1^, whereas the full cell without mPEG‐P only achieves 28%. In addition, this work renders new perspectives through comprehensive characterizations such as small‐angle X‐ray scattering to reveal the distinctive solvation/surface structures and the importance of spatial ordering regulation for improving the performance of zinc batteries. Therefore, this study offers deep insights into the electrolyte design and interfacial/interphase characterizations, aiming to enhance energy efficiency and durability of AZIBs for practical applications.

## Conflict of Interest

The authors declare no conflict of interest.

## Author Contributions

G.C. and J.L. conceived and supervised the project. Z.Y. and J.L. designed and performed the experiments. Z.Y., G.C., and J.L. contributed to data analysis. Y.S. carried out the calculations. Z.Y., G.C., and J.L. wrote the paper. All authors discussed and revised the manuscript.

## Supporting information

Supporting Information

## Data Availability

The data that support the findings of this study are available from the corresponding author upon reasonable request.
