# Supplementary material for: Noncovalent Interactions‐Driven Self‐Assembly of Polyanionic Additive for Long Anti‐Calendar Aging and High‐Rate Zinc Metal Batteries
Source: Adv Sci (Weinh). 2024 Jun 27;11(33):2404513. doi: 10.1002/advs.202404513 (PMC11434035; doi:10.1002/advs.202404513)
Supplement: Supplementary file 1 — Supporting Information [file ADVS-11-2404513-s001.docx]

**Supporting Information**

Noncovalent interactions driven self-assembly of polyanionic additive for long-term anti-calendar aging and high-rate zinc metal batteries

Zimin Yang,^1,3^ Yilun Sun,^1^ Jianwei Li,^*2^ Guanjie He,^4^ Guoliang Chai^*1,5^

^1^ State Key Laboratory of Structural Chemistry, Fujian Institute of Research on the Structure of Matter, Chinese Academy of Sciences, Fuzhou, 350002 Fujian, P. R. China.

^2^ Key Laboratory of Comprehensive and Highly Efficient Utilization of Salt Lake Resources, Qinghai Province Key Laboratory of Resources and Chemistry of Salt Lakes, Qinghai Institute of Salt Lakes, Chinese Academy of Sciences, Xining, Qinghai 810008, P. R. China.

^3^ College of Chemistry and Materials Science, Fujian Normal University, Fuzhou 350007, Fujian, P. R. China.

^4^ Christopher Ingold Laboratory, Department of Chemistry, University College London, London, WC1H 0AJ, UK

^5^ School of Chemical Science, University of Chinese Academy of Sciences, Beijing 100049, China.

KEYWORDS: aqueous Zn-ion batteries, calendar aging, electrolyte additive, ion transfer, self-assemble

Corresponding Author Email: Jianwei.li@isl.ac.cn; [g.chai@fjirsm.ac.cn](mailto:g.chai@fjirsm.ac.cn)

**Experimental**

**Materials**

Methoxy polyethylene glycol-phosphate (mPEG-P, Mw=350, liquid), Ammonium metavanadate (NH_4_VO_3_, AR), N-methyl pyrrolidone (NMP, 99.5%) were purchased from Aladdin. Zn(OTf)_2_ was purchased from TCI. Ethanedioic acid dihydrate (H_2_C_2_O_4_·2H_2_O, AR) was purchased from Sinopharm Chemical Reagent Co., Ltd. Conductive carbon black (EC600JD) and polyvinylidene fluoride (PVDF, ≥99.5%) were purchased from Arkema. MnO_2_ (99.95%) was purchased from Macklin. Zinc foils (0.05 mm thickness, 99.999% purity) and Cu foils (0.05 mm thickness, 99.99% purity) were purchased from Guangdong Ares Metal Technology Co. Ltd.

**Synthesis of NH_4_V_4_O_10_ material**

0.585 g of NH_4_VO_3_ was introduced to 30 mL of deionized water, followed by stirring for a duration of 10 minutes. Subsequently, 0.18 g of H_2_C_2_O_4_·2H_2_O was slowly added, with stirring sustained until a dark blue-green solution was procured. The above solution was then transported into a 50 mL autoclave and placed in an oven at 180 °C for 6 hours. The obtained material was collected, thoroughly washed with deionized water, and then freeze-drying over a period of 2 days.

**Fabrication of electrolytes**

mPEG-P was mixed with deionized water in different proportions by volume (mPEG-P/H_2_O) = 0, 0.5, 1 and 2%). Then, 2m Zn(OTf)_2_ was dissolved into the mPEG-P-H_2_O co-solvents to prepare the 0, 0.5%, 1%, and 2% mPEG-P electrolytes, respectively.

**Characterizations**

The Nuclear Magnetic Resonance (NMR, JEOL ECZ400S, Japan), Fourier transform infrared (FTIR, Thermo Nicolet iS50), and Confocal *In Situ* Raman Spectroscopy (LabRAM HR Evolution) were employed to analyze the element and surface chemistry of the samples. The ionic conductivity was determined by a conductivity meter (DDS-11A, INESA Scientific Instrument Co. Ltd., China). The Zeta potentials were collected using Laser Particle Size and Zeta Potential Analyzer (BI-200SM BROOKHAVEN). The SAXS and GISAXS data were obtained by the small-angle X-ray scattering instrument (Xeuss 3.0). Zn foils were characterized with X-ray diffraction (XRD, MiniFlex 600, Cu-K_α_ radiation), Scanning Electron Microscopy (SEM, Hitachi SU-8010), and X-ray photoelectron spectroscopy (XPS, ESCALAB 250Xi spectrophotometer with Al‐K radiation system). The *in-situ* optical microscope images were obtained on an electron microscope (XJ-906H, Shenzhen Xianjian Juye Electronics Co. Ltd., China) by using a homemade *in-situ* optical electrochemical cell. Atomic force microscopy (AFM, [Bruker Dimension Icon](http://bruker.cnreagent.com/product_1792.html)) and Stylus Profiler (Dektak XT) were carried out to study the electrode surface. The contents of dissolved manganese elements in different electrolytes were tested by Inductively Coupled Plasma Optical Emission Spectrometer (ICP-OES, Avio220Max). The SAXS data was obtained by the small-angle X-ray scattering instrument (Xeuss 3.0). The *in-situ* generation of hydrogen was tested by Differential Electrochemical Mass Spectrometer (DEMS) (QAS100 Li, Linglu Instruments Co.,Lt). The X-ray absorption fine structure analysis (XAFS) experiment described in this paper was performed at the Shanghai Synchrotron Radiation Facility.

**Electrochemical Measurements**

Zn plating/stripping properties of Zn symmetric batteries and electrochemical performance of full batteries were performed with CR2025 coin-type cells. The performance of coin-type cells was recorded on NEWARE battery-testing instrument (BTS-5V10/20/50mA/1A, Shenzhen, China) at room temperature. The cathode was fabricated by mixing NH_4_V_4_O_10_ /the commercial MnO_2_ powder, super P (EC600JD), and PVDF with a weight ratio of 7:2:1 using N-methyl pyrrolidone (NMP) as solvent. The mixture slurry was printed on hydrophilic carbon paper and then transferred to a vacuum oven drying under 60℃ for 12 h. Finally, cathodes were prepared by cutting circular discs from the dried samples. The loading mass of the cut disk of electrodes is all within the scope of 2.1~2.7 mg along with a diameter of 1.2 cm. The loading mass of active materials of electrodes is all within the scope of 1.3~ 1.7 mg cm^-2^. Zinc foil (0.05 mm thickness) and Whatman GF/C glass microfiber were employed as the anode and separator, respectively. Linear sweep voltammetry (LSV), cyclic voltammetry (CV), chronoamperometry (CA), and electrochemical impedance spectroscopy (EIS) were performed using an electrochemical workstation (CHI760E, Shanghai, China). The LSV test was conducted using a three-electrode system, with Ag/AgCl electrode, glass carbon electrode, and Ti anode serving as the reference, counter, and working electrodes, respectively. CV curves of Zn plating/stripping using Ti as the working electrode and Zn as the reference and counter electrode. CA plots were carried out with Zn symmetric batteries (coin-type cells) at an overpotential of -150 mV and a duration of 400 s. According to the previous report, the EDLC value was calculated by the following equation:

*C*= *i*/*v* (S1)

Where *C* is capacitance, *i* is current. The *i* was defined by half of the difference between positive and negative scanning current at each scanning rate. EIS was measured for Zn//Zn symmetric cells with different electrolytes over a frequency range of 0.01 Hz to 100 kHz and fitted using ZView2 Analysis software.

The transference number of (t_Zn_^2+^) was calculated by the following equation (the typical Evans method):

(S2)

Where *∆V* is the applied polarization voltage (15 mV), *I_0_* and *R_0_* are the initial current and resistance. *I_s_* and *R_s_* are the steady-state current and resistance, respectively.

**Computational Methods**

The calculations of the binding energies, HOMO and LUMO were performed with Gaussian 16 program^[1]^. The geometric structure of the molecules and ions were calculated by M06-2X^[2]^ method with a basis set aug-cc-pVTZ.^[3]^ The vibrational frequencies and thermal correction to Gibbs free energy of the molecules and ions were calculated at optimized geometry using the same level of theory. The energies of binding energies are Gibbs free energies.The electrostatic potential (ESP) was created by GaussView 6.0 from total SCF density. The method and basis set used in the calculation are identical to those mentioned above.

The ﬁrst-principles DFT calculations of adsorption energies were performed using the Vienna *Ab initio* Simulation Package (VASP) code.^[4]^ All the calculations were carried out by using Perdew−Burke−Ernzerh (PBE) for exchange−correlation functional. The spin-polarized Kohn−Sham equations were solved in the plane-wave and pseudopotential framework with a cutoﬀ of 500 eV. Gamma points were used for all the DFT calculations.


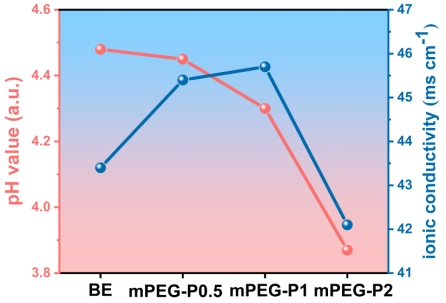


**Figure. S1** Measured pH and ionic conductivity of the electrolytes.


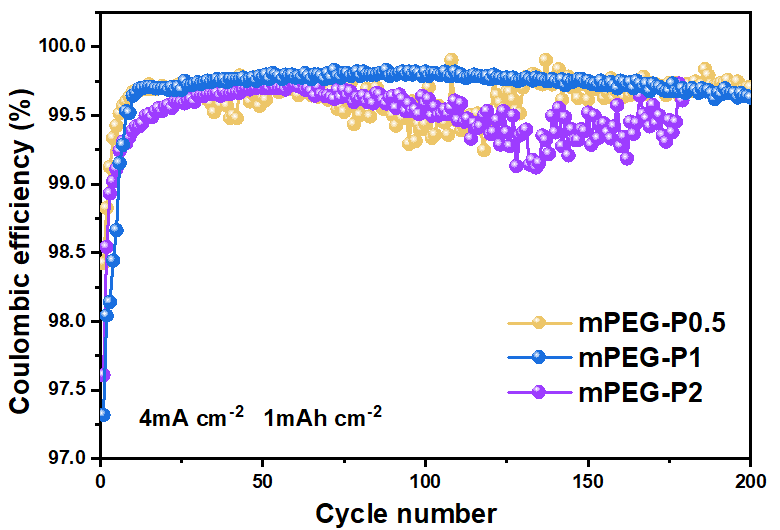


**Figure. S2** Coulombic efficiency of Zn/Cu cells in electrolytes with different content of mPEG-P additives.


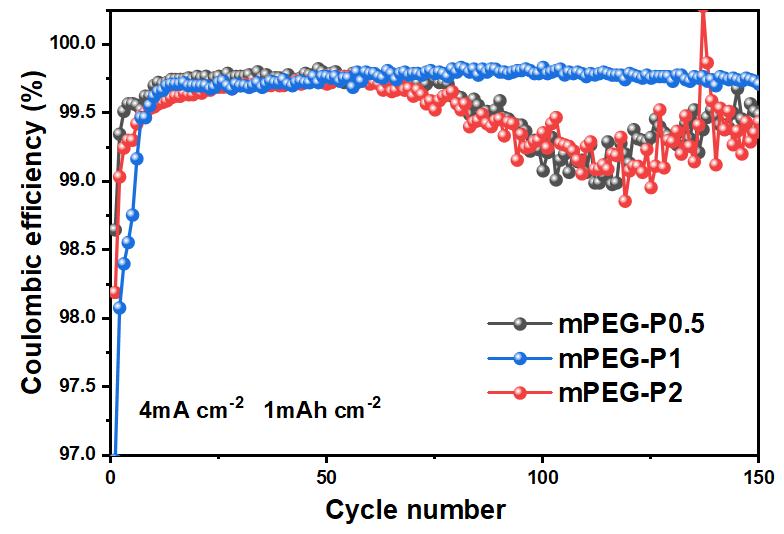


**Figure. S3** Reproduced Coulombic efficiency test of Zn/Cu cells in electrolytes with different content of mPEG-P additives.


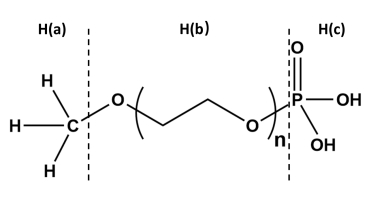


**Figure. S4** The structural formula of mPEG-P (n=5-6).


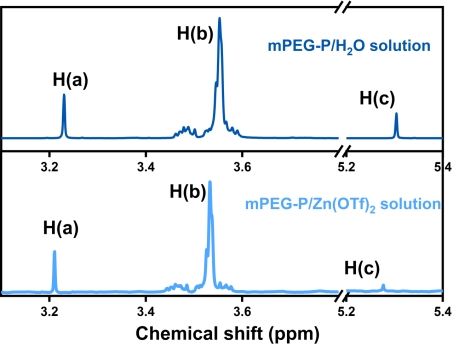


**Figure. S5** ^1^H NMR of the different electrolytes.


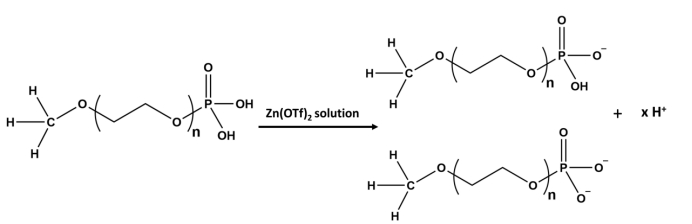


**Figure. S6** The illustration of the deprotonation process of mPEG-P.


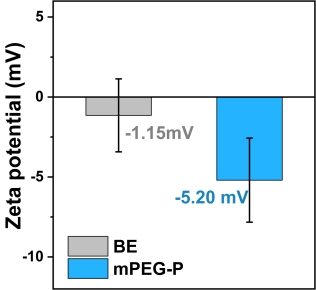


**Figure. S7** The Zeta potential of different electrolytes.


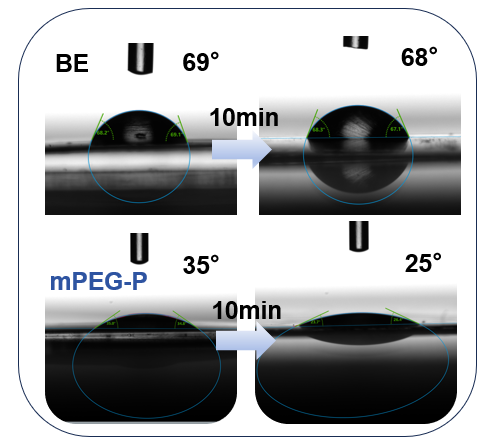


**Figure. S8** The contact angles of the different electrolytes using bare zinc foil as the substrate.


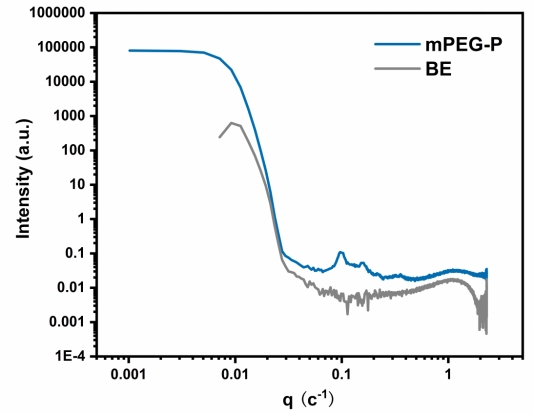


**Figure. S9** Small angle X-ray scattering scans of different electrolytes.

**Figure. S10 (a)** Small angle X-ray scattering curve of mPEG-P electrolyte and lamellae model layer; (b) Small angle X-ray scattering curve of parametric analysis of layer structures.


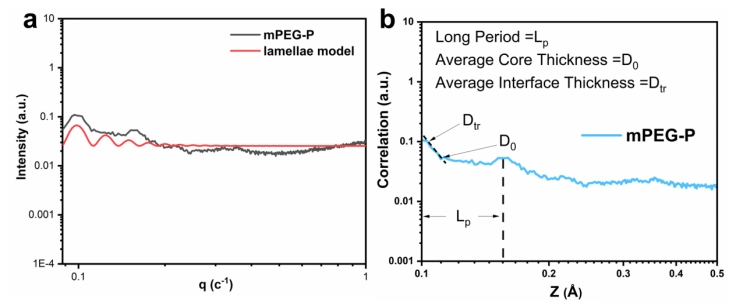

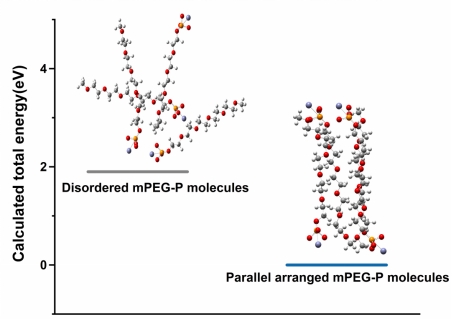


**Figure. S11** The relative energy of two different optimized structures containing four mPEG-P molecules. The energy of parallel arranged mPEG-P molecules is set to 0 eV as reference.


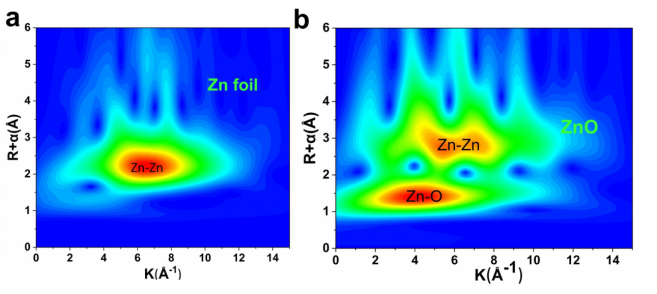


**Figure. S12** Wavelet transform images of the EXAFS spectra for a) Zn foil and b) ZnO, respectively.


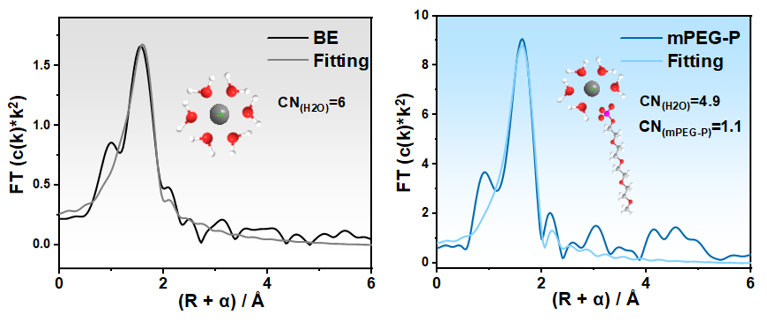


**Figure. S13** FT-EXAFS fitting curves at R space of different electrolytes (CN: coordination numbers).


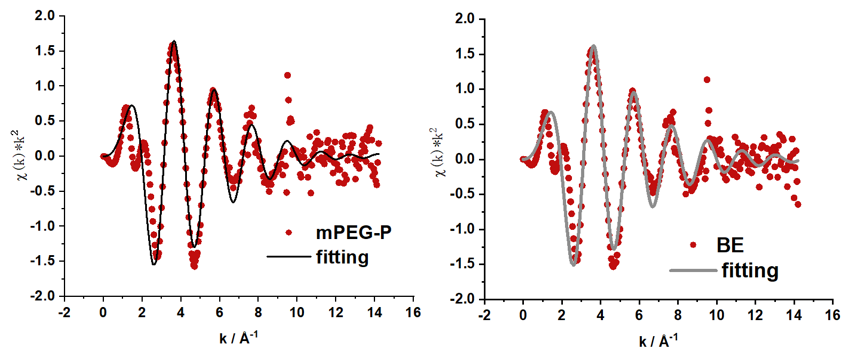


**Figure.S14** K^2^ -weighted Fourier transform of Zn K-edge EXAFS spectra of different electrolytes in the k-space.


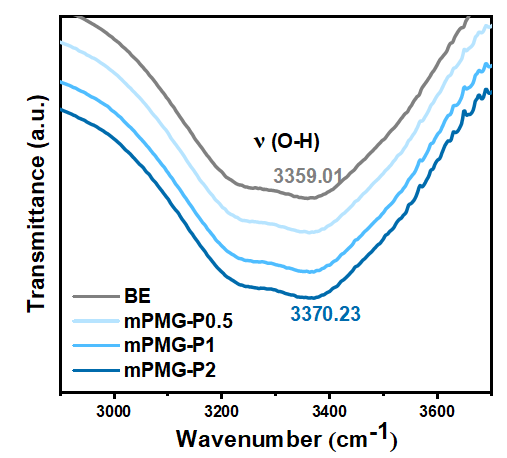


**Figure. S15** FTIR spectra of the electrolytes containing different concentrations of mPEG-P.


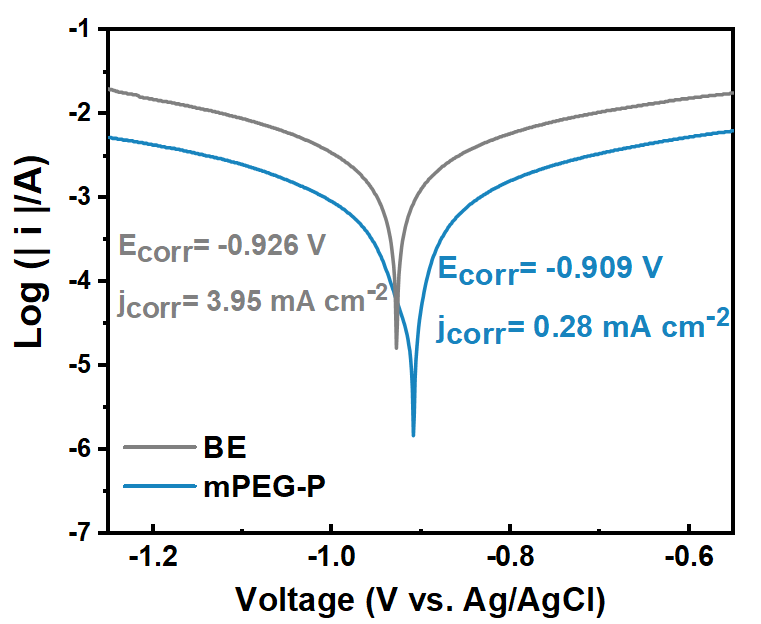


**Figure. S16** Linear polarization curves for describing corrosion of Zn anodes in different electrolytes.


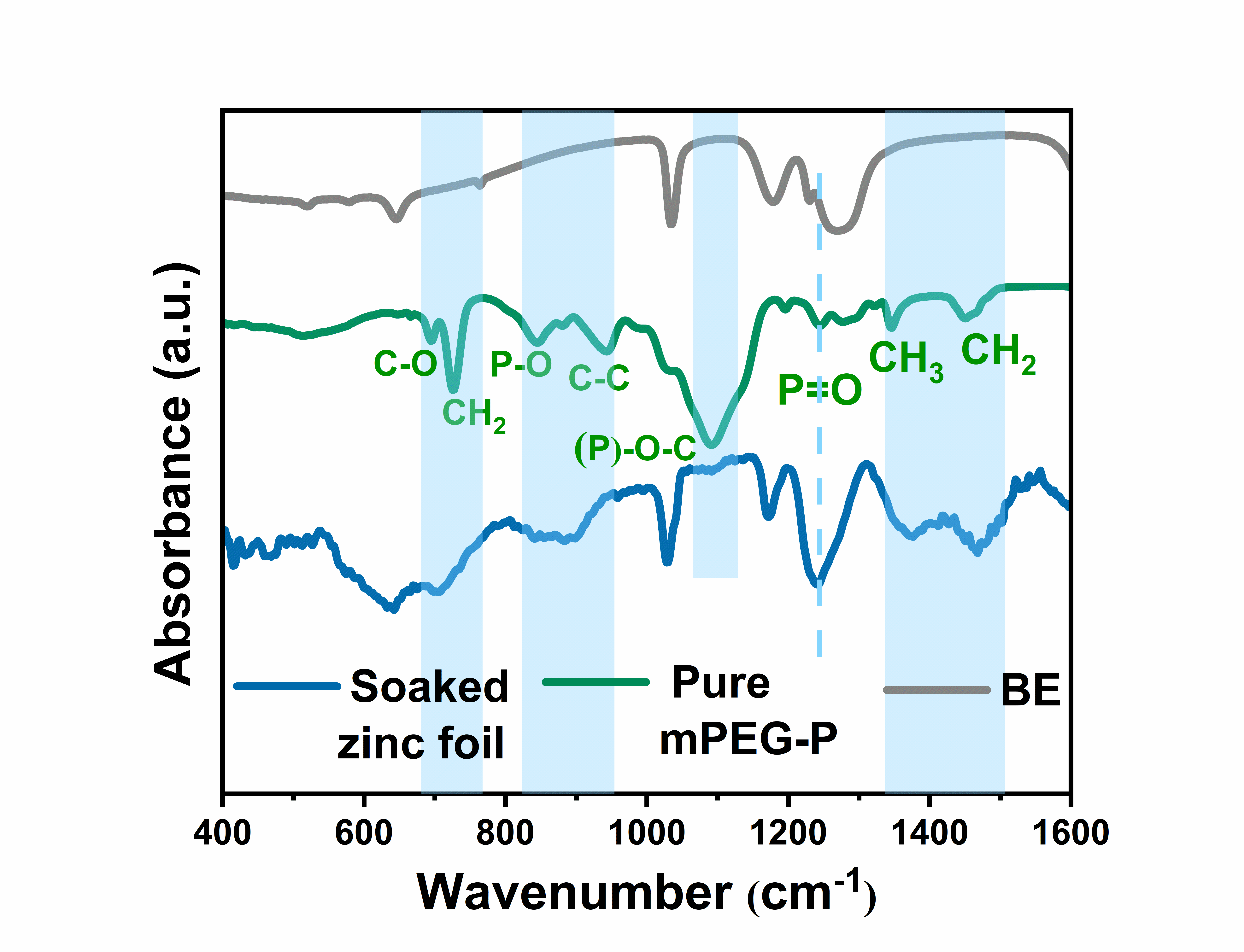


**Figure. S17** The FTIR spectra of different electrolytes and soaked zinc foils.


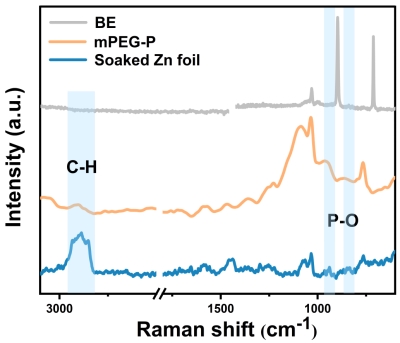


**Figure. S18** Raman spectra of different electrolytes and soaked Zn foil.


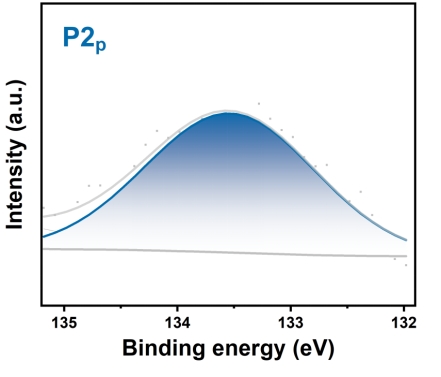


**Figure. S19** The core-level P 2*p* spectra of Zn foils after soaking in the mPEG-P/Zn(OTf)_2_ electrolyte for 4 hours.


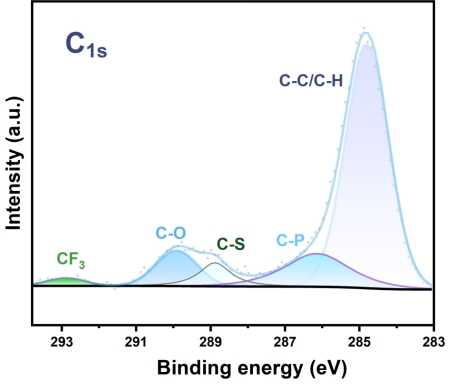


**Figure. S20** The core-level C 1*s* spectra of Zn foils after soaking in the mPEG-P/Zn(OTf)_2_ electrolyte for 4 hours.


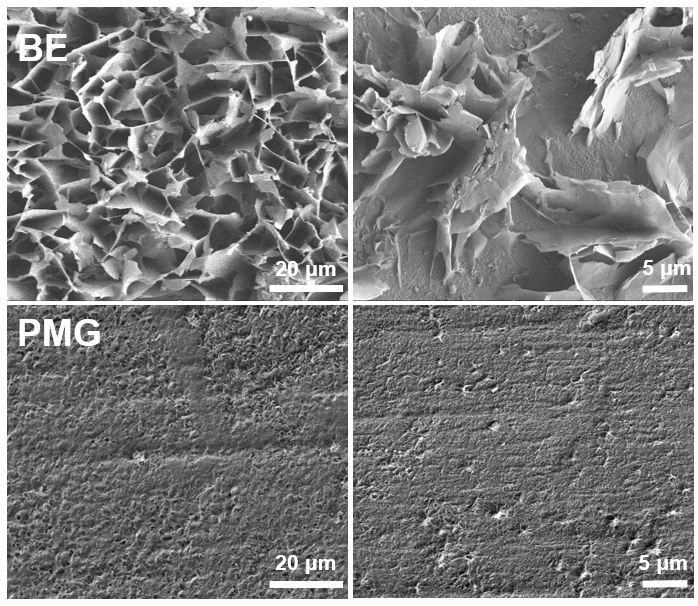


**Figure. S21** SEM images of Zn foils after soaking in different electrolytes for 4 h.


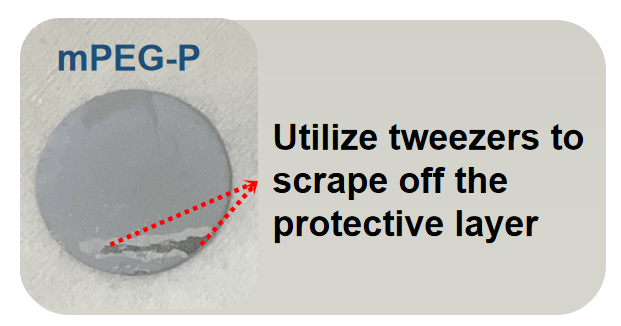


**Figure S22** Description of the surface treatment of zinc foils after 30 days of immersion in mPEG-P electrolyte.


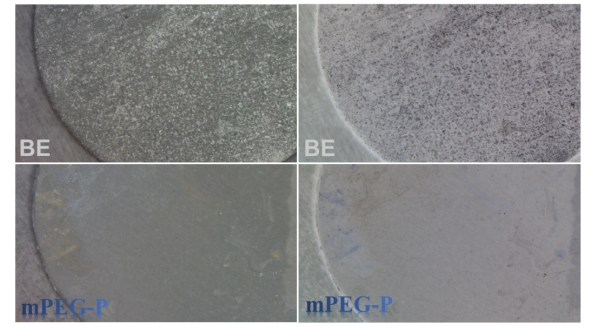


**Figure. S23** Electron microscope images of Zn foils after soaking in different electrolytes for 30 days.


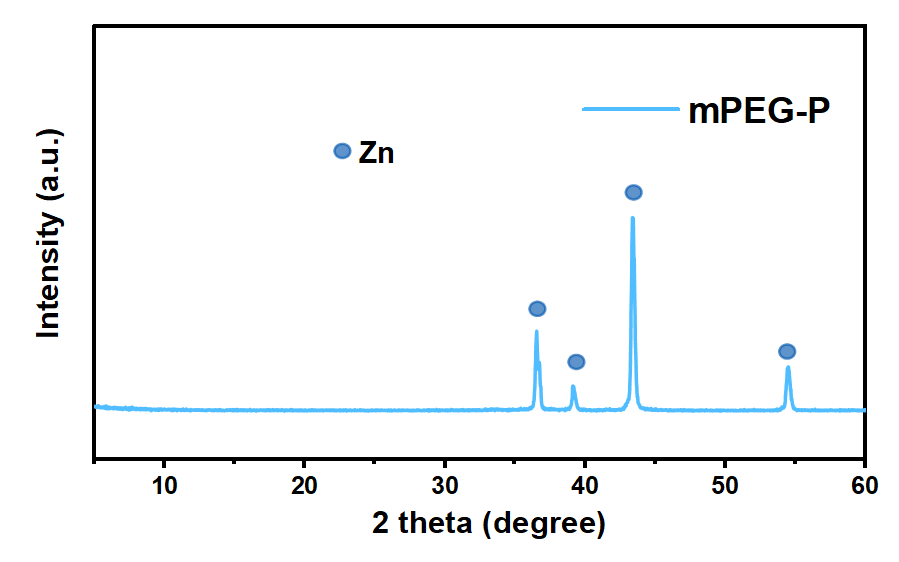


**Figure S24** XRD patterns of Zn foils after soaking in mPEG-P aqueous solution for 7 days.


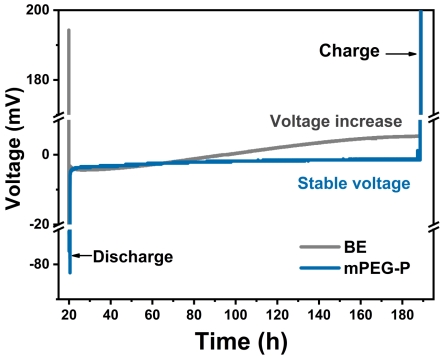


**Figure. S25** Voltage changes of Zn/Cu cells during resting in 7 days.


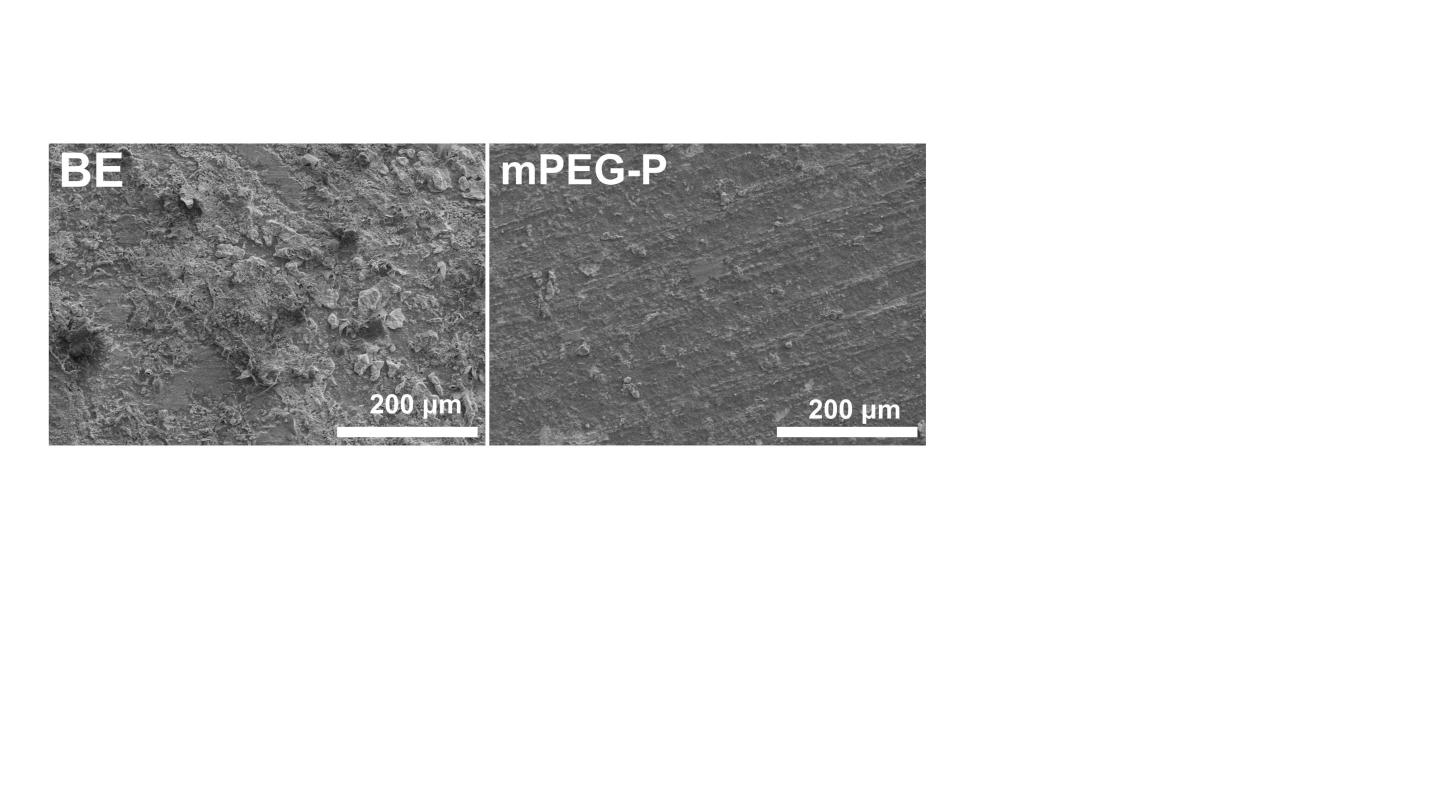


**Figure S26** SEM images of Zn foils in Zn//Cu batteries with different electrolytes after 7 days of aging.


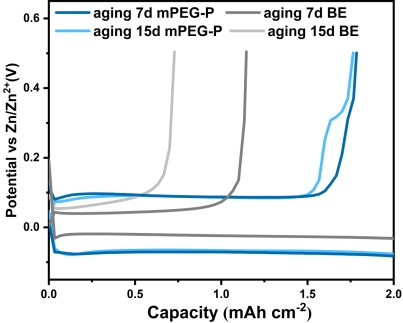


**Figure. S27** The corresponding voltage profiles of cells aged for 7/15 days in electrolytes with/without mPEG-P.


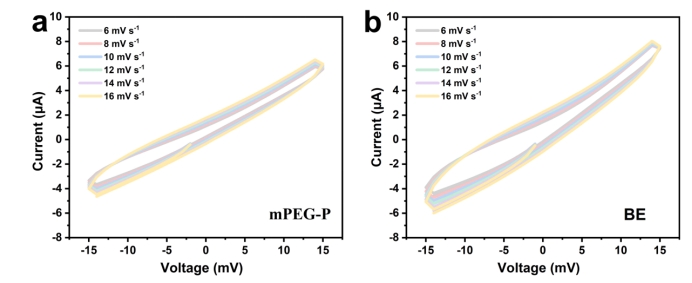


**Figure. S28** Cyclic voltammograms curves for Zn//Zn symmetric coin cells with (a) and without (b) mPEG-P additive in a voltage range from -15 mV to 15 mV under various scanning rates.


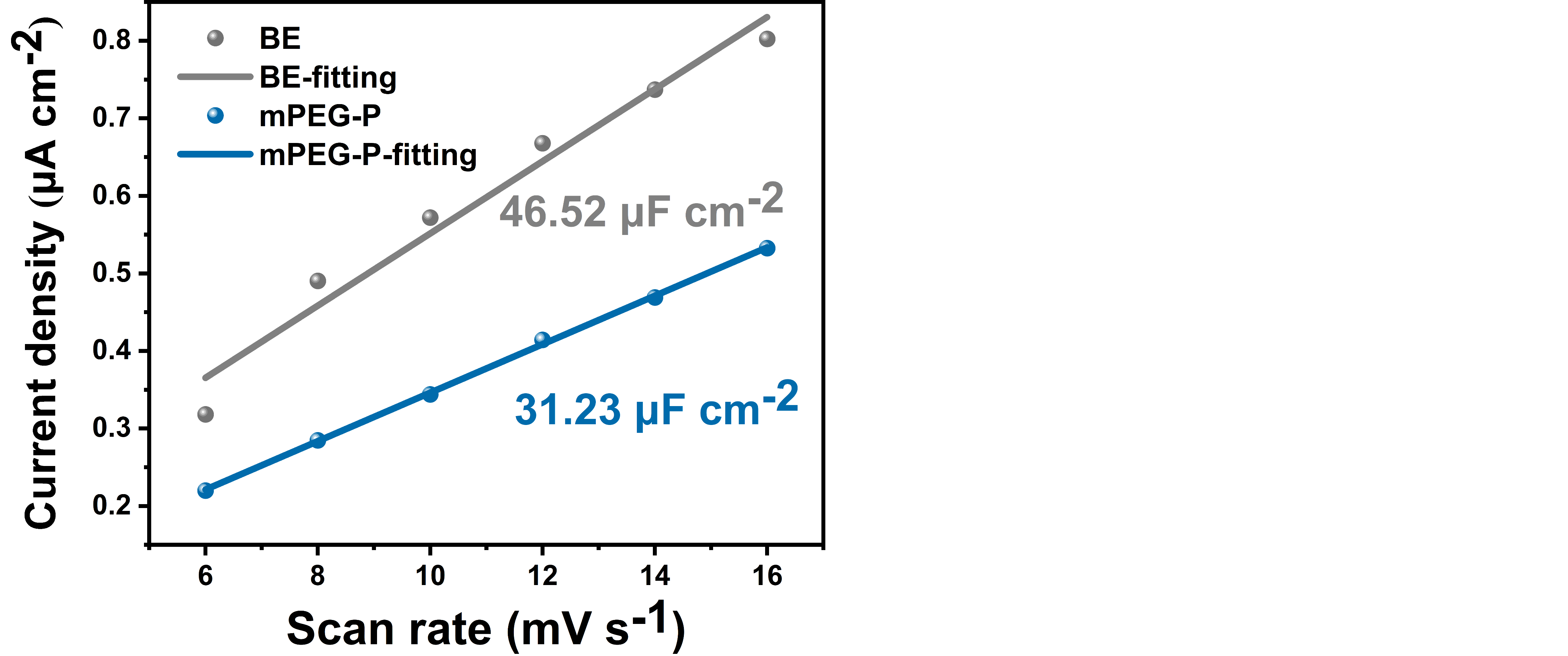


**Figure. S29** EDL measurements for Zn anodes in electrolytes with/without mPEG-P additive.


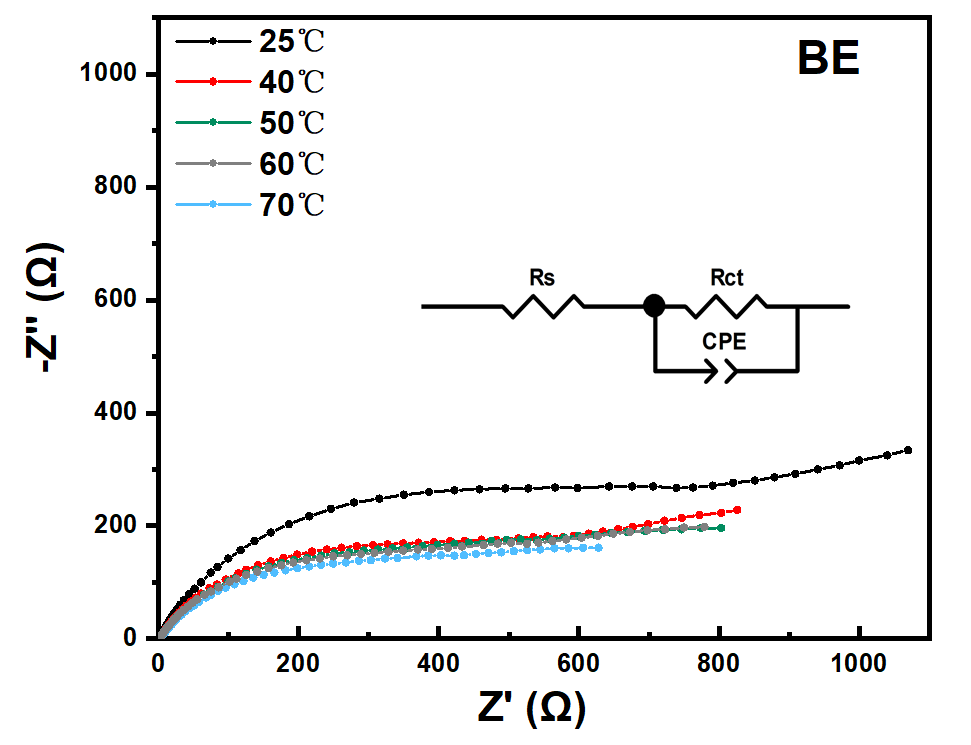


**Figure. S30** Nyquist plots at different temperatures for Zn//Zn symmetrical batteries in pure Zn(OTf)_2_ electrolyte (the insert picture is the fitting circuit of symmetric batteries).

The Arrhenius activation energy (*E_a_*) can be approximated as the desolvation energy barrier for hydrated Zn^2+^, which can be calculated according to the following Arrhenius equation^5^:

1/*R_ct_* = A exp (*E_a_*/RT) (S3)

where *R_ct_*, A, R, and T represent the charge-transfer resistance, the frequency factor, the gas constant, and the absolute temperature, respectively.


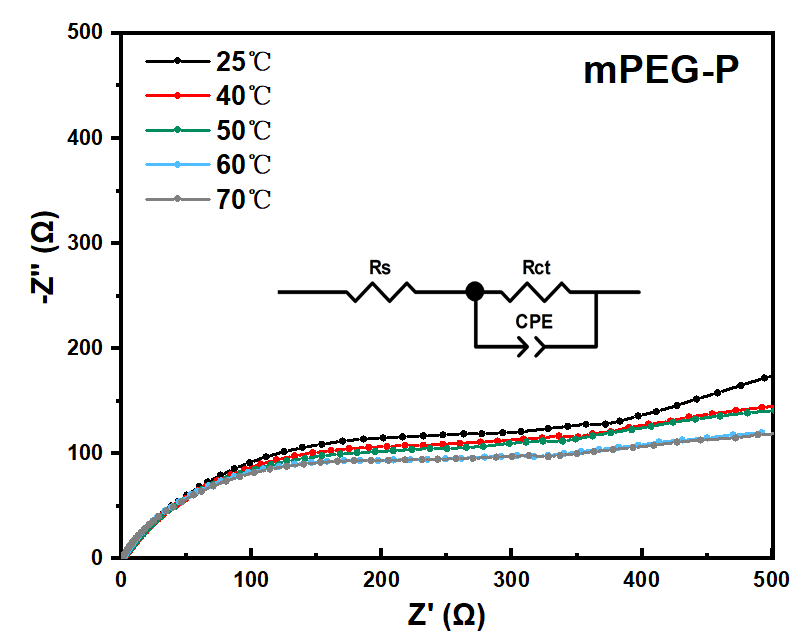


**Figure. S31** Nyquist plots at different temperatures for Zn//Zn symmetrical batteries in mPEG-P/Zn(OTf)_2_ electrolyte (the insert picture is the fitting circuit of symmetric batteries).


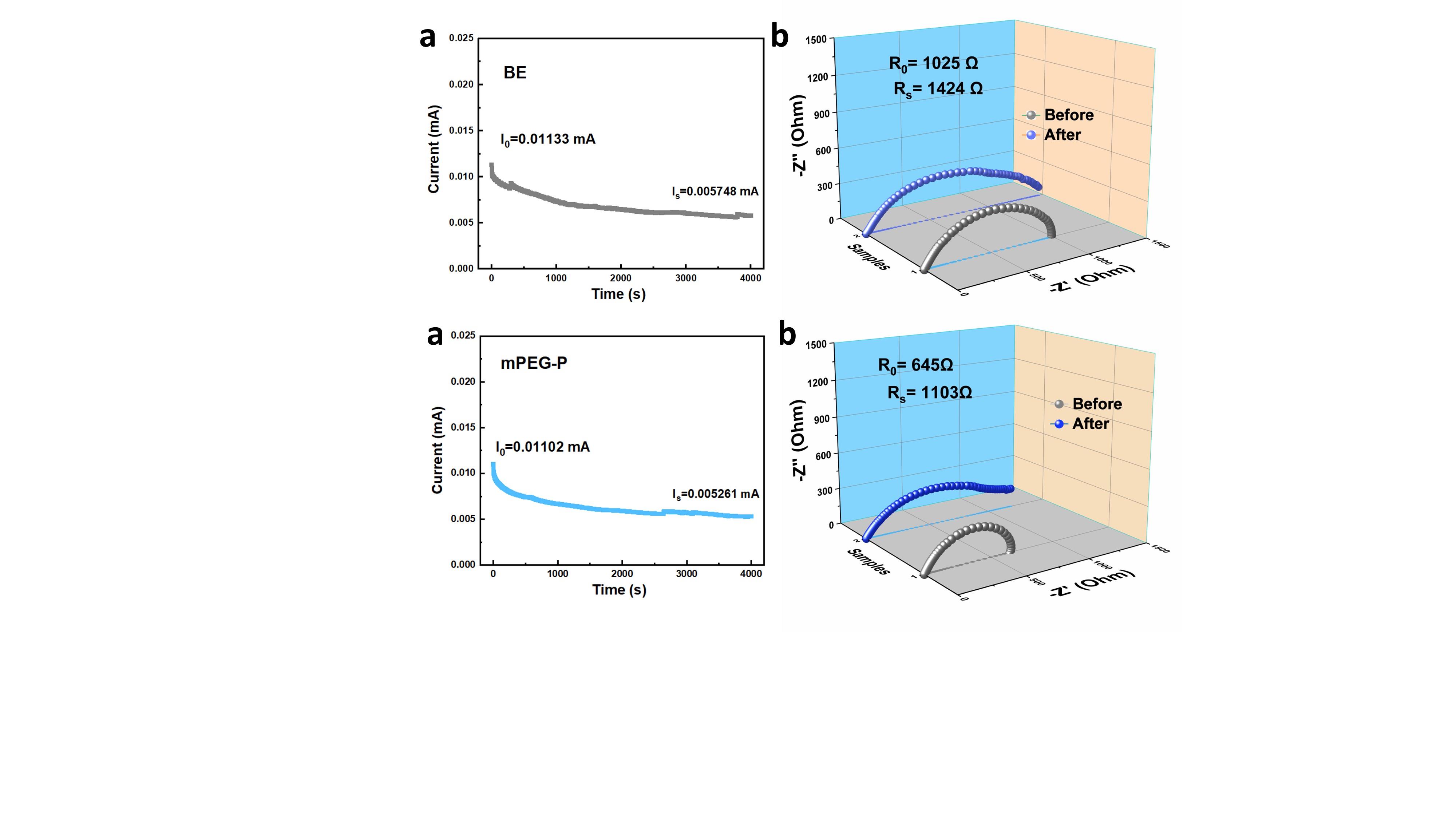


**Figure. S32** (a) Current-time plots of Zn//Zn symmetric cells with Zn(OTf)_2_ electrolyte after polarization at a constant potential (15 mV) for 4000 s. (b) The impedance spectra before and after polarization.


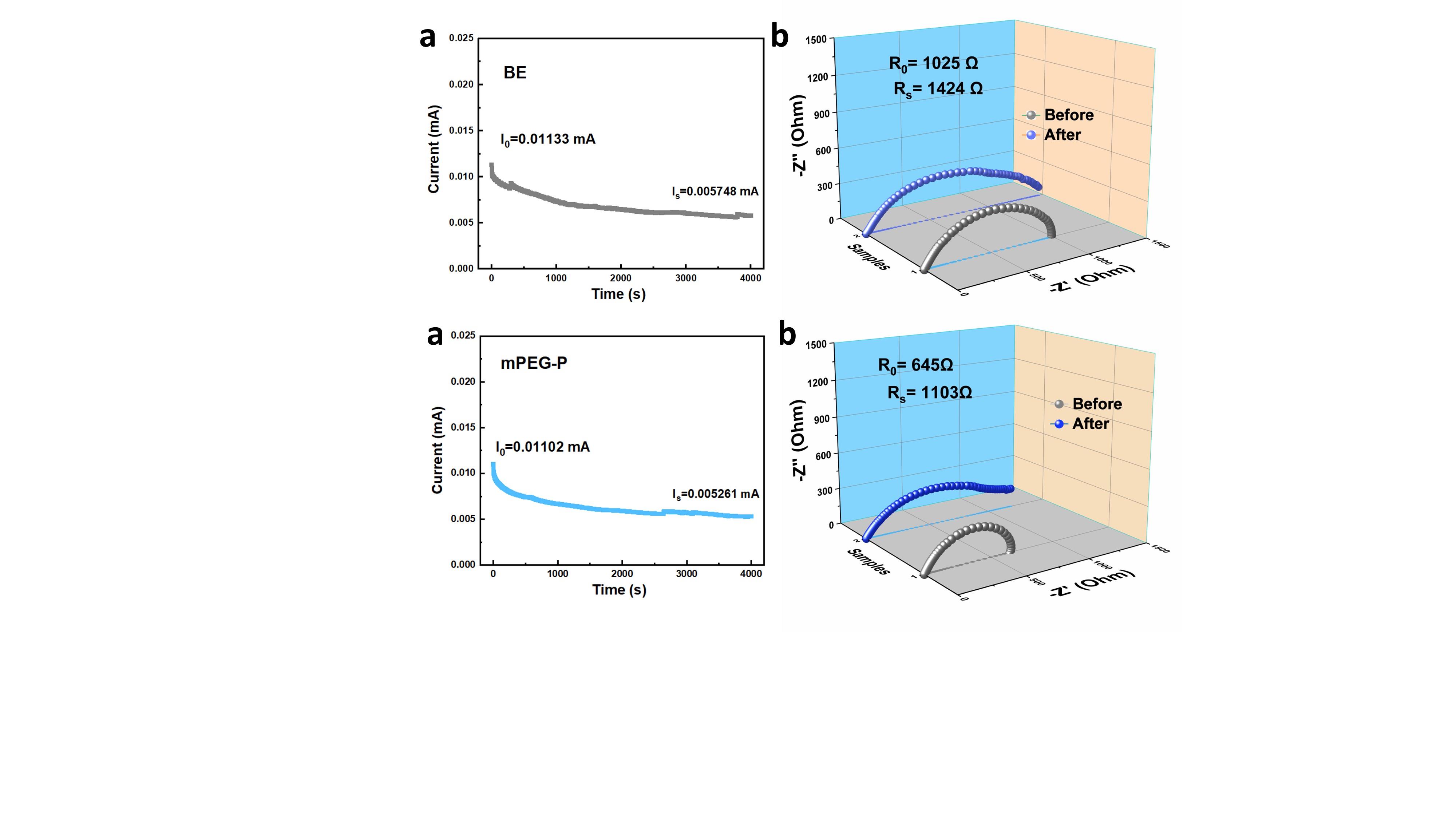


**Figure. S33** (a) Current-time plots of Zn symmetric cells with mPEG-P/Zn(OTf)_2_ electrolyte after polarization at a constant potential (15 mV) for 4000 s. (b) The impedance spectra before and after polarization.


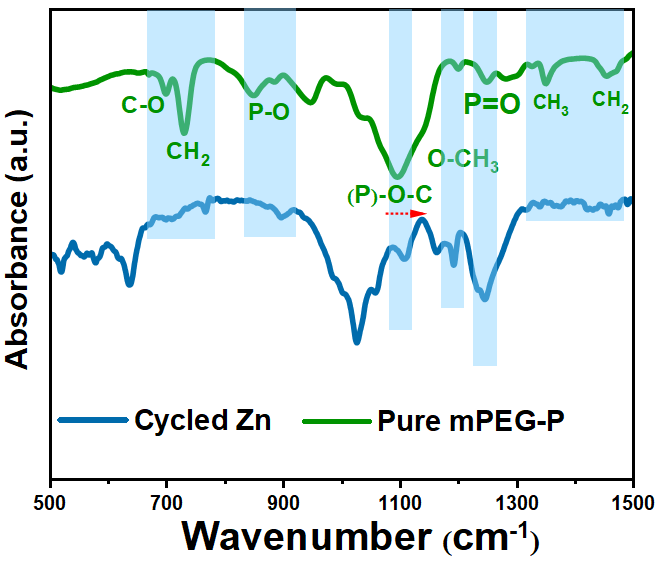


**Figure. S34** FTIR spectra of mPEG-P and cycled Zn anode.


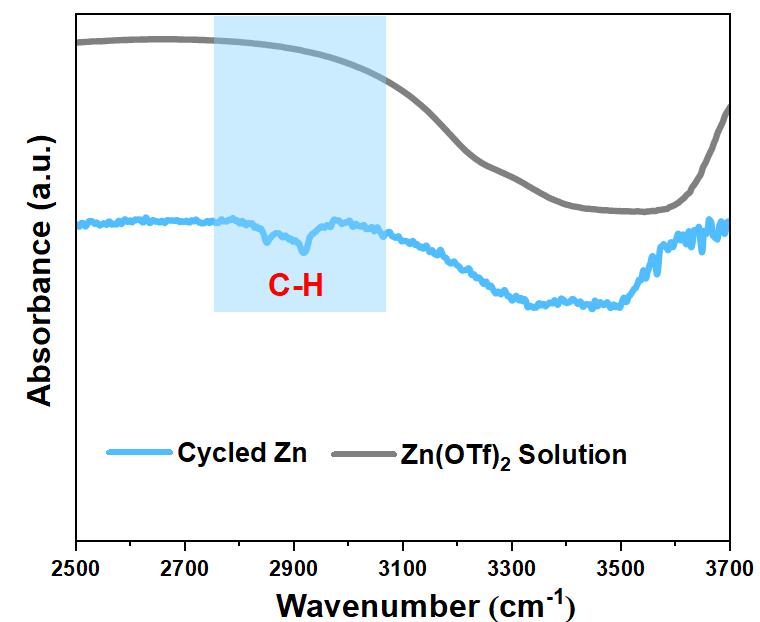


**Figure. S35** FTIR spectra of the Zn(OTf)_2_ electrolyte and cycled Zn anode.


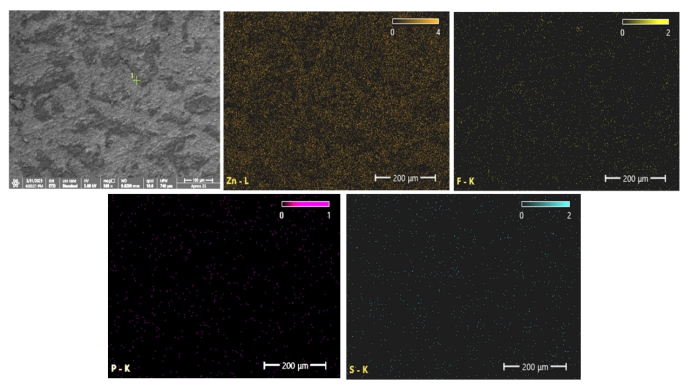


**Figure. S36** SEM image and EDS elemental mapping of the Zn anode after cycling for 110 hours.


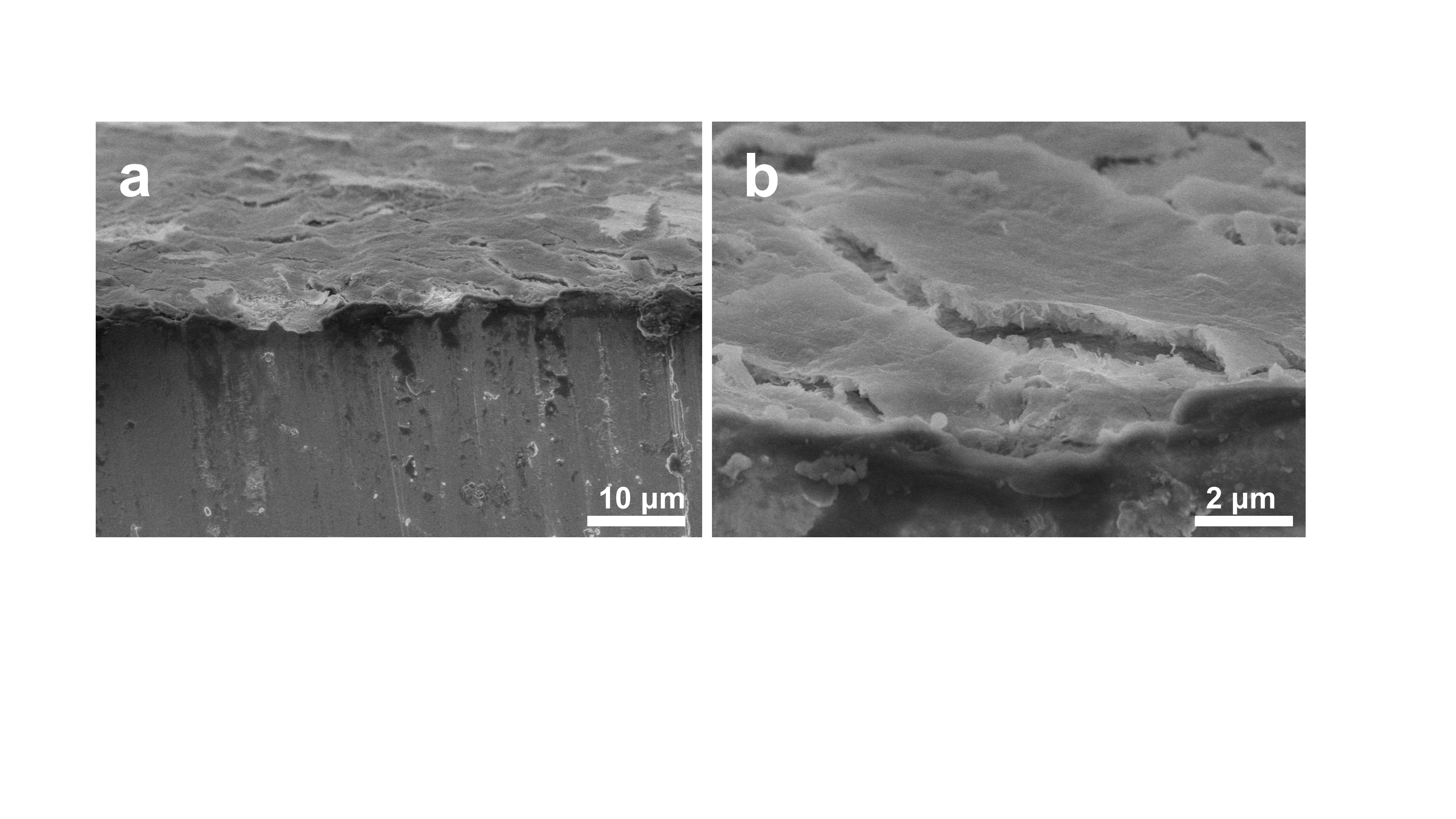


**Figure. S37** The SEM images of the Zn anode in mPEG-P/Zn(OTf)_2_ electrolyte.


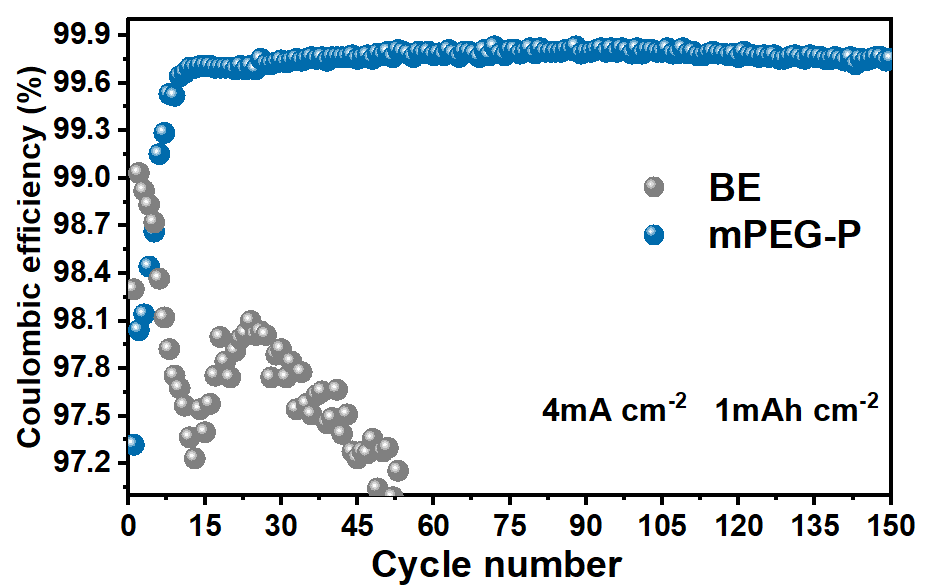


**Figure. S38** CE and cycling performance of Zn//Cu cells under current density of 4 mA cm^−2^ with 1 mAh cm^−2^.


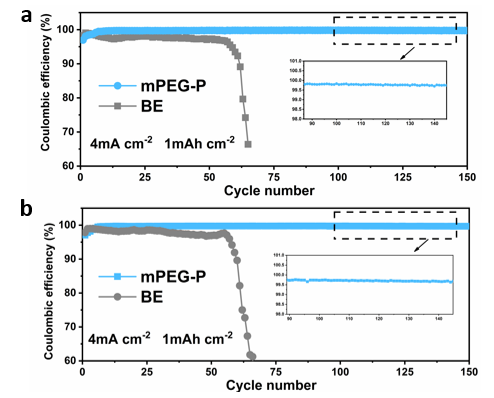


**Figure. S39** Reproduced CE and cycling performance of Zn//Cu cells under current density of 4 mA cm^−2^ with 1 mAh cm^−2^.


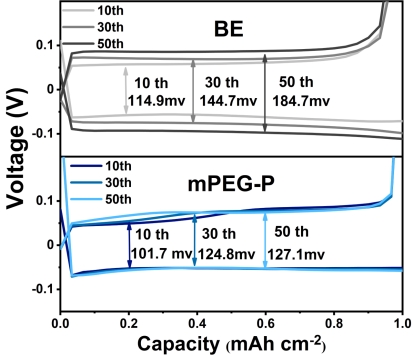


**Figure. S40** Voltage profiles of Zn//Cu cells in electrolytes with/without mPEG-P additive.


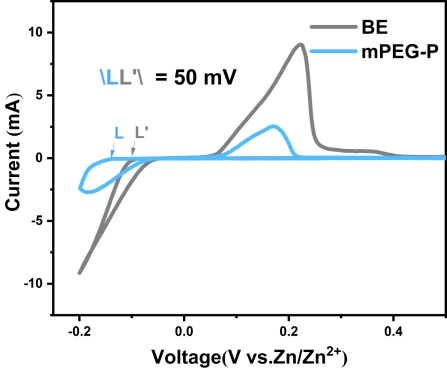


**Figure. S41** CV curves of Zn/Ti cells in electrolyte with/without mPEG-P additive.


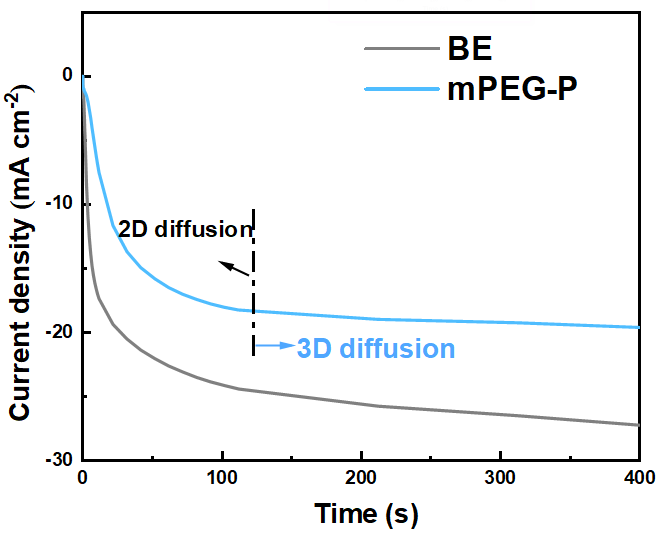


**Figure. S42** Chronoamperometry curves of Zn//Zn symmetric cells with different electrolytes at an overpotential of -150 mV.


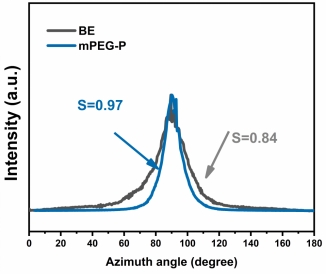


**Figure. S43** The azimuth angle of different electrolytes.


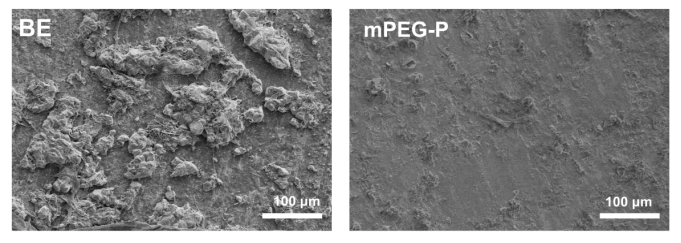


**Figure. S44** The images of Zn anodes in Zn(OTf)_2_ electrolyte and mPEG-P/Zn(OTf)_2_ electrolyte after cycling for 100 h.


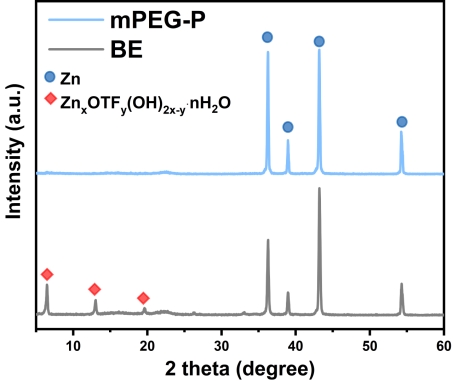


**Figure. S45** XRD patterns of Zn anodes in different electrolytes after cycling for 50 hours.


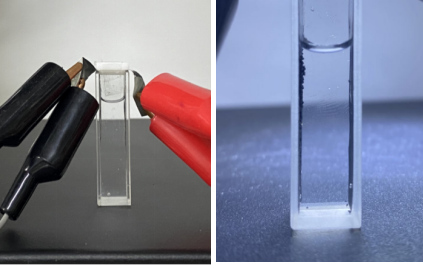


**Figure. S46** A homemade optical electrochemical cell for *in-situ* microscope observation.


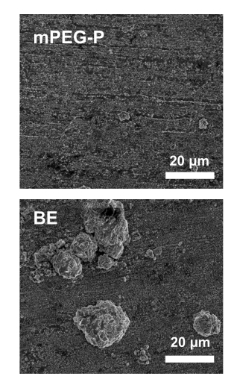


**Figure. S47** The SEM images of cycled Zn foils in the *in-situ* optical electrochemical cells.


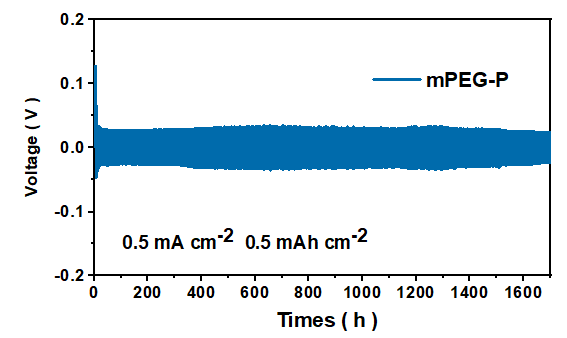


**Figure. S48** Reproduced long-term galvanostatic cycling test with mPEG-P additive at 0.5 mA cm^-2^ and 0.5 mAh cm^-2^.


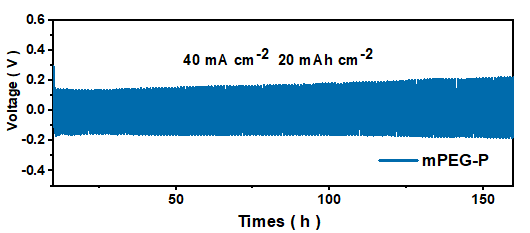


**Figure. S49** Reproduced long-term galvanostatic cycling test with mPEG-P additive at 40 mA cm^-2^ and 20 mAh cm^-2^.


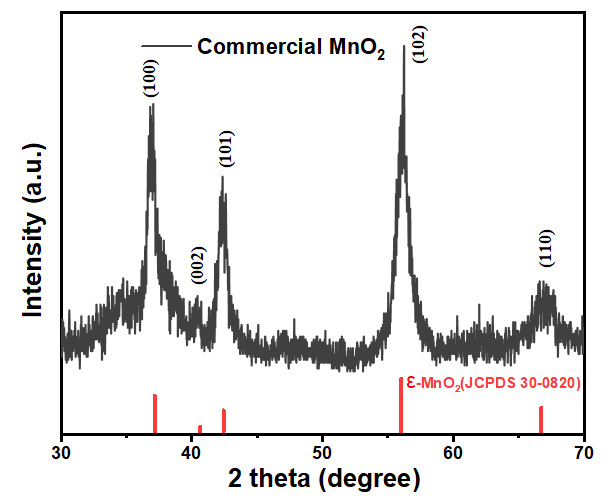


**Figure. S50** XRD patterns of commercial MnO_2_ powder.


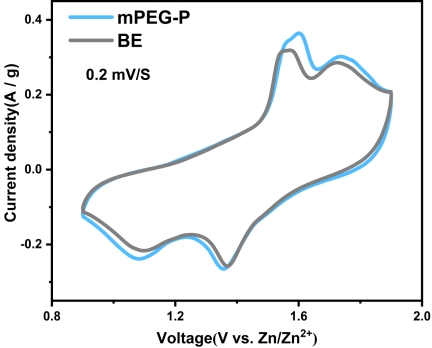


**Figure. S51** Cyclic voltammetry curves of the full cells in different electrolytes.


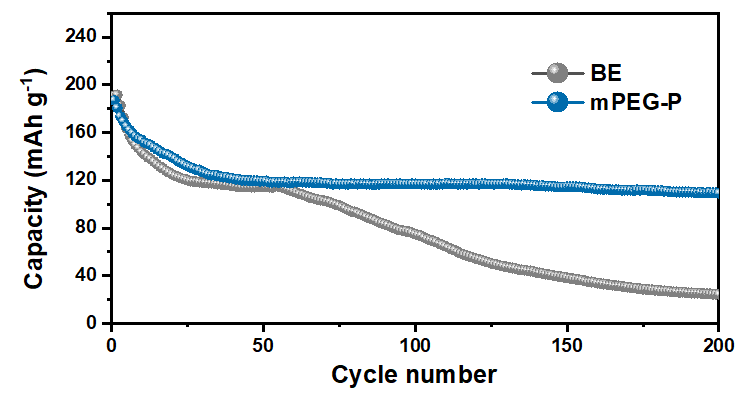


**Figure. S52** Reproduced cycling performance at a current density of 0.2 A g^-1^


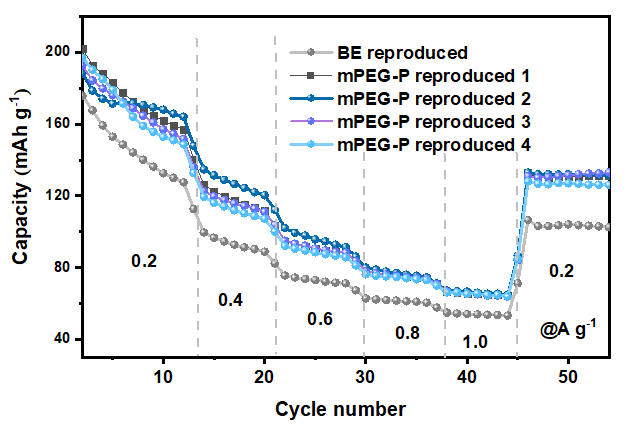


**Figure. S53** Reproduced comparison of capacities of full cells at various current densities.


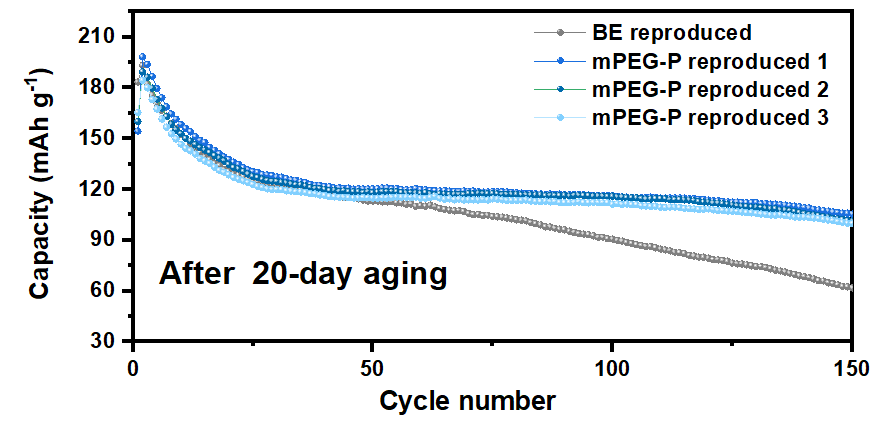


**Figure. S54** Reproduced cycling performance at a current density of 0.2 A g^-1^ after 20-day aging process.


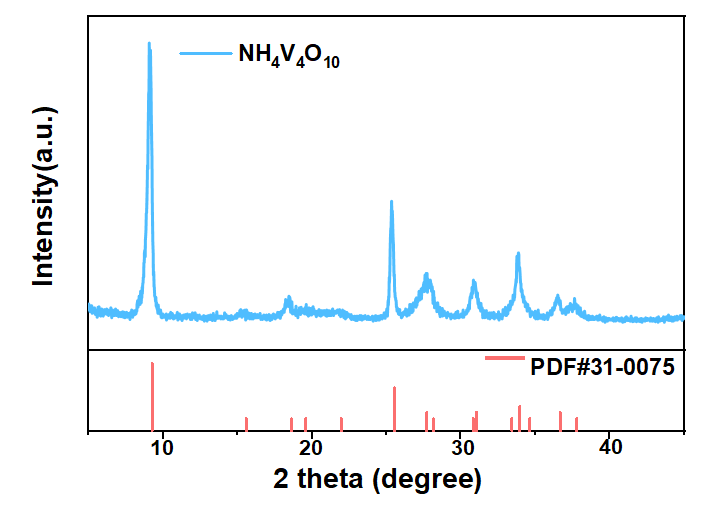


**Figure. S55** XRD pattern of the as-prepared NH_4_V_4_O_10_.

\


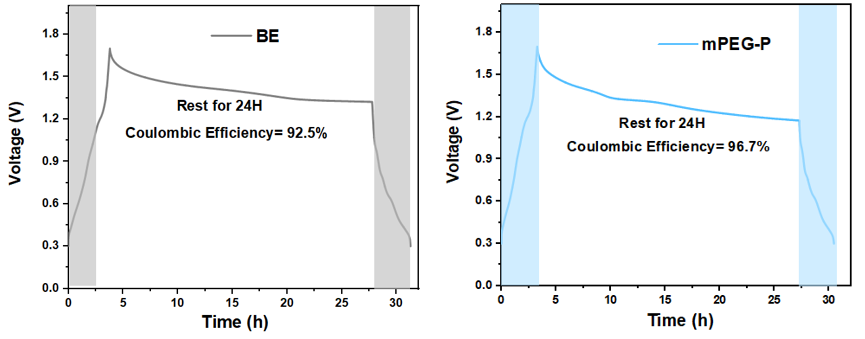


**Figure. S56** Self-discharge curves of full cells in electrolytes with different electrolytes.


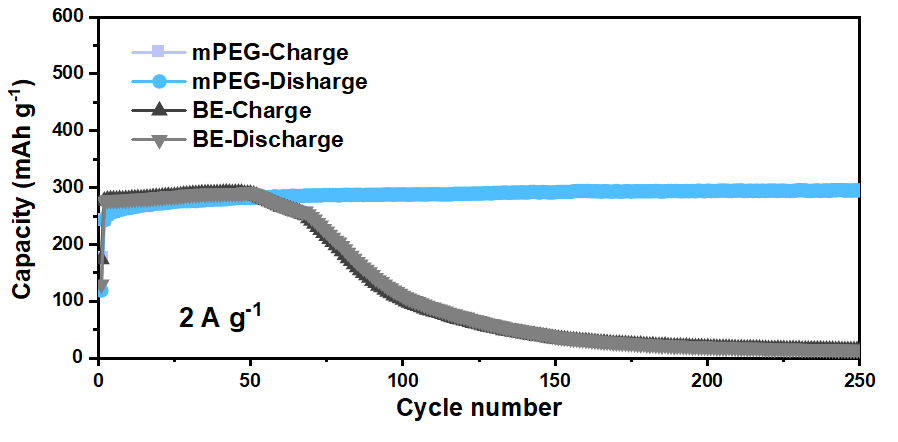


**Figure. S57** Comparison of the cycling performances of Zn//NVO batteries at 2 A g^-1^


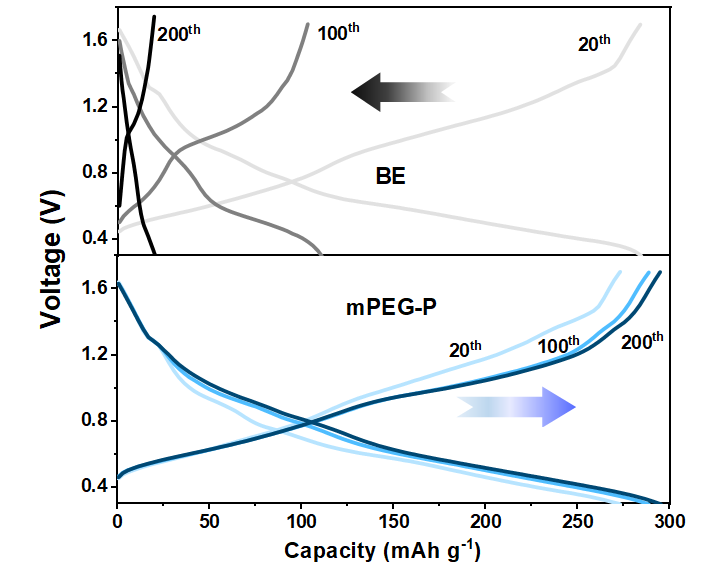


**Figure. S58** The charge-discharge profiles of Zn//NVO with the BE electrolyte and mPEG-P electrolyte.

Table S1. Fitting results of EXAFS of different samples

| Sample | Path | CN | R(Å) | σ^2^(10^-3^Å^2^) | ΔE_0_(eV) | R factor |
| --- | --- | --- | --- | --- | --- | --- |
| **BE** | Zn-O | 6.0 | 2.06 | 7.0 | 1.10 | 0.021 |
| **mPEG-P** | Zn-O (C) | 1.1 | 1.96 | 1.5 | 0.076 | 0.014 |
|  | Zn-O (H) | 4.9 | 2.09 | 5.0 | 0.076 | 0.014 |

CN: coordination numbers: R: bond distance: σ^2^: Debye-Waller factors; ΔE_0_: the inner potential correction; R factor: goodness of fit. S_0_^2^ was set as 0.96. The data ranges are presented as 3 ≤ k ≤ 12.7 Å^-1^, 1.1 ≤ R ≤ 2.7 Å for BE; 3 ≤ k ≤ 12.27 Å^-1^, 1 ≤ R ≤ 2.8 Å for mPEG-P. Error bounds that characterize the structural parameters obtained by EXAFS spectroscopy were estimated as CN ± 20%: σ^2^ ± 20%: R ± 0.03 Å

Table S2. Fitting results for symmetric cells at different temperatures.

| Symmetrical cells | Res | 25℃ | 40℃ | 50℃ | 60℃ | 70℃ |
| --- | --- | --- | --- | --- | --- | --- |
| mPEG-P/Zn(OTf)_2_ electrolyte  Zn(OTf)_2_ electrolyte | R_ct_  R_ct_ | 368.9  796.8 | 336.5  608.5 | 316.9  555 | 294.1  498.6 | 279.4  433.7 |

Table S3. Performance of Zn//Zn symmetric cells using mPEG-P/Zn(OTf)_2_ electrolyte comparied with other reported literatures.

| No. | Zn//Zn symmetric cell design | Current density  (mA cm^-2^) | Capacity  (mAh cm^-2^) | Cumulative plated capacity (Ah cm^-2^) | Reference |
| --- | --- | --- | --- | --- | --- |
| **1** | **mPEG-P/Zn(OTf)_2_** | **20**  **40** | **20**  **40** | **6**  **3.6** | **This**  **work** |
| 2 | CN membranes | 10 | 2 | 1.2 | 6 |
| 3 | ZP coated | 20 | 1 | 0.775 | 7 |
| 4 | Zn/IHS electrode | 20 | 20 | 1.6 | 8 |
| 5 | Pectin/ZnSO_4_ | 5 | 5 | 2.25 | 9 |
| 6 | Zn-ILL layer | 20 | 10 | 2.5 | 10 |
| 7 | FCOF@Zn | 40 | 1 | 0.75 | 11 |
| 8 | ESA/ZnSO_4_ | 20 | 2 | 1.5 | 12 |
| 9 | ZP@Zn | 20 | 5 | 2.35 | 13 |
| 10 | β-Cd/ZnSO_4_ | 40 | 20 | 1.7 | 14 |

**References**

[1] Risch, M. J.; Trucks, G. W.; Schlegel, H. B.; Scuseria, G. E.; Robb, M. A.; Cheeseman, J. R.; Scalmani, G.; Barone, V.; Petersson, G. A.; Nakatsuji, H.; Li, X.; Caricato, M.; Marenich, A.V; Bloino, J.; Janesko, B. G.; Gomperts, R.; Mennucci, B.; Hratchian, H. P.; Ortiz, J.V; Izmaylov, A. F.; Sonnenberg, J. L.; Williams-Young, D.; Ding, F.; Lipparini, F.; Egidi, F.; Goings, J.; Peng, B.; Petrone, A.; Henderson, T.; Ranasinghe, D.; Zakrzewski, V. G.; Gao, J.; Rega, N.; Zheng, G.; Liang, W.; Hada, M.; Ehara, M.; Toyota, K.; Fukuda, R.; Hasegawa, J.; Ishida, M.; Nakajima, T.; Honda, Y.; Kitao, O.; Nakai, H.; Vreven, T.; Throssell, K.; Montgomery Jr., J. A.; Peralta, J. E.; Ogliaro, F.; Bearpark, M. J.; Heyd, J. J.; Brothers, E. N.; Kudin, K. N.; Staroverov, V. N.; Keith, T. A.; Kobayashi, R.; Normand, J.; Raghavachari, K.; Rendell, A. P.; Burant, J. C.; Iyengar, S. S.; Tomasi, J.; Cossi, M.; Millam, J. M.; Klene, M.; Adamo, C.; Cammi, R.; Ochterski, J. W.; Martin, R. L.; Morokuma, K.; Farkas, O.; Foresman, J. B.; Fox, D. J. Gaussian16 Revision B.01. **2016**.

[2] Y. Zhao, D. G. Truhlar, *Theor. Chem. Acc.* **2008**, *120*, 215-241.

[3] a) T. H. Dunning, Jr., *J. Chem. Phys.* **1989**, *90*, 1007-1023; b) R. A. Kendall, T. H. Dunning, Jr., R. J. Harrison, *J. Chem. Phys.* **1992**, *96*, 6796-6806.

[4] a) G. Kresse, J. Hafner, *Phys. Rev. B.* **1994**, *49*, 14251-14269; b) G. Kresse, J. Furthmüller, *Comp. Mater. Sci.* **1996**, *6*, 15-50.

[5] X. Yang, C. Li, Z. Sun, S. Yang, Z. Shi, R. Huang, B. Liu, S. Li, Y. Wu, M. Wang, Y. Su, S. Dou and J. Sun, *Adv. Mater.*, **2021**, 33, 2105951.

[6] Y. Li, X. Peng, X. Li, H. Duan, S. Xie, L. Dong, F. Kang, *Adv. Mater.* **2023**, *35*.

[7] H. J. Kim, S. Kim, K. Heo, J. H. Lim, H. Yashiro, S. T. Myung, *Adv. Energy Mater.* **2022**, 13.

[8] Z. Cai, Y. Ou, B. Zhang, J. Wang, L. Fu, M. Wan, G. Li, W. Wang, L. Wang, J. Jiang, Z. W. Seh, E. Hu, X.-Q. Yang, Y. Cui, Y. Sun, *J. Am. Chem. Soc.* **2021**, *143*, 3143-3152.

[9] L. Hong, J. Guan, Y. Tan, Y. Chen, Y.-S. Liu, W. Huang, C. Yu, Y. Zhou, J.-S. Chen, K.-X. Wang, *Energy Environ. Sci*., **2024**,17, 3157-3167.

[10] J. Ji, Z. Zhu, H. Du, X. Qi, J. Yao, H. Wan, H. Wang, L. Qie, Y. Huang, *Adv. Mater.* **2023**, *35*.

[11] Z. Zhao, R. Wang, C. Peng, W. Chen, T. Wu, B. Hu, W. Weng, Y. Yao, J. Zeng, Z. Chen, P. Liu, Y. Liu, G. Li, J. Guo, H. Lu, Z. Guo, *Nat. Commun.* **2021**, *12*.

[12] Y. Wang, R. Zhao, M. Liu, J. Yang, A. Zhang, J. Yue, C. Wu, Y. Bai, *Adv. Energy Mater*. 2023, **13**, 2302707.

[13] Z. Xing, Y. Sun, X. Xie, Y. Tang, G. Xu, J. Han, B. Lu, S. Liang, G. Chen, J. Zhou, *Angew. Chem. Int. Ed.* **2022**, *62*.

[14] C. Meng, W. He, L. Jiang, Y. Huang, J. Zhang, H. Liu, J. J. Wang, *Adv. Funct. Mater.* **2022**, *32*.
